# Genome expression profile analysis of the maize sheath in response to inoculation to *R. solani*

**DOI:** 10.1007/s11033-014-3103-z

**Published:** 2014-01-14

**Authors:** Jian Gao, Zhe Chen, Mao Luo, Hua Peng, Haijian Lin, Cheng Qin, Guangsheng Yuan, Yaou Shen, Haiping Ding, Maojun Zhao, Guangtang Pan, Zhiming Zhang

**Affiliations:** 1 Key Laboratory of Biology and Genetic Improvement of Maize in Southwest Region, Ministry of Agriculture, Maize Research Institute of Sichuan Agricultural University, Wenjiang, 611130 Sichuan China; 2 Drug Discovery Research Center of Luzhou Medical College, Luzhou, 646000 Sichuan China; 3 Life Science College of Sichuan Agricultural University, Ya’an, 625014 Sichuan China

**Keywords:** DGE (Solexa digital gene expression), Maize sheath tissues, qRT-PCR quantitative real-time PCR, TPM transcript per million, BLSB

## Abstract

Currently, the molecular regulation mechanisms of disease-resistant involved in maize leaf sheaths infected by banded leaf and sheath blight (BLSB) are poorly known. To gain insight into the transcriptome dynamics that are associated with their disease-resistant, genome-wide gene expression profiling was conducted by Solexa sequencing. More than four million tags were generated from sheath tissues without any leaf or development leaf, including 193,222 and 204,824 clean tags in the two libraries, respectively. Of these, 82,864 (55.4 %) and 91,678 (51.5 %) tags were matched to the reference genes. The most differentially expressed tags with log2 ratio >2 or <−2 (*P* < 0.001) were further analyzed, representing 1,476 up-regulated and 1,754 down-regulated genes, except for unknown transcripts, which were classified into 11 functional categories. The most enriched categories were those of metabolism, signal transduction and cellular transport. Next, the expression patterns of 12 genes were assessed by quantitative real-time PCR, and it is showed the results were general agreement with the Solexa analysis, although the degree of change was lower in amplitude. In conclusion, we first reveal the complex changes in the transcriptome during the early development of maize sheath infected by BLSB and provide a comprehensive set of data that are essential for understanding its molecular regulation mechanism.

**Electronic supplementary material:**

The online version of this article (doi:10.1007/s11033-014-3103-z) contains supplementary material, which is available to authorized users.

## Introduction

*Rhizoctonia solani Kühn* is a plant pathogenic fungus with a wide host range and worldwide distribution and has a significant economic impact in the development and production of a wide variety of crops. To date, 13 Anastomosis Groups (AGs) have been recognized according to hyphal anastomosis behavior, cultural morphology, host range and pathogenicity [54]. Furthermore, the variability in the degree of hyphal fusion, morphology, pathogenicity and host range observed in AGs (AG-1, AG-13) and AG-1A has been widely endanger to maize as a dominant fungus in southwest of China and identified as anastomosis group [60].

Banded leaf and sheath blight (BLSB), caused by *R. solani Kühn*, is one of the most important diseases of maize (*Zea mays.)* worldwide. Severe yield losses can result as a consequence of the rapid development and large-scale spread of the disease epidemic under optimal environmental conditions. Futhermore, *R. solani Kühn* is a common soil borne pathogen with a great diversity of host plants that can attack previously resistant cultivars, along with the capacity of fungal spores to travel long distances, making control of BLSB difficult [61]. And this disease initially infects maize at the first and second leaf sheath above the ground and then spreads upward to infect the ear, leading to severe yield losses [61]. Recently, yield losses approached 100 % in southern of China when the ear rot phase predominated [27].

 Over the last few years, genetic and molecular studied on the disease and pathogen have been reported in maize [36, 39, 62]. These studies have revealed that resistance to BLSB is a typical quantitative trait controlled by polygenes and three significant QTL located on chromosomes two, six, and ten to be responsible for resistance to BLSB respectively [8, 13, 61]. In addition, many catalytic enzymes involved in response to BLSB infection were analyzed, including chitinase, glucanase and phenylanine ammonia lyase [32, 35] and few pathogenesis-associated genes and some potential defense pathways were involved in response to BLSB infection [2, 64]. However, the use of most genetic and molecular techniques in studying genes involved in the maize-BLSB interactions has been largely limited due to the BLSB that is hard to produce any asexual (conidia) spores although it is considered to have an asexual life cycle.

To understand the mechanism(s) of the host resistance at the molecular level, a critical first step is to identify the transcripts that accumulate in response to the pathogen attack. In this study, “R15”, a identified inbred lines with good general combining ability, good agronomic characteristics and high level of resistance to BLSB [59], was employed to identify a set of candidate genes associated with BLSB resistance using Solexa’s Digital Gene Expression (DGE) technology. Which is upgraded from the previous massively parallel signature sequencing (MPSS) technology, [7, 29, 42] can produce the specific 3′signature for each mRNA, thereby reducing library saturation from abundant transcripts and enhancing the capacity for rare transcript detection [4, 43]. This study will help to elucidate the molecular mechanism of resistance to BLSB and provide important evidence for breeding excellent maize lines.

## Materials and methods

### Plants material and pathogen infection

High-resistance maize inbred line seedlings of “R15” were treated with 7 % hypochlorite solution for 30 min respectively, followed by three washes with sterilized water before being sowed in pots with autoclaved soil. Control plants were maintained under the same conditions. *R. solani AG1*-*IA* was cultured on potato dextrose agar and incubated at 28 °C for three days before use (Support information Figure S1). Infections were conducted by inoculating with *R. solani* (kindly provided by the Rice Institution of Sichuan Agricultural University, Sichuan, China) at 28 °C after growing for 35 days. The leave sheaths were covered with plastic bags to ensure high humidity. The leaf sheaths without any leaves were harvested from each of three maize plants, and the three leaf sheaths were combined to represent one replicate. Three independent replicates were collected for each sample. Infected leave sheaths were collected every 12 h for 2 days. Control samples were harvested from water-treated leaf sheaths incubated under the same conditions. (Support information Figure S2).

### Preparation of digital expression libraries

Samples from infected leaf sheaths from 36 to 48 h were pooled for RNA isolation and library construction. Comparable control leaf sheaths were treated identically and in parallel. Sequence tag preparation was done with the Digital Gene Expression Tag Profiling Kit according to the manufacturer’s protocol. 20 μg of total RNA and 6 μg of mRNA were obtained and purified by adsorption of biotin Oligo(dT) magnetic beads. After mRNA’s binding, cDNA synthesis was performed. Double strand cDNA was introduced into cDNA fragment digested by NlaIII endonuclease and these binging fragment containing sequences of CATG site and adjacent polyA tail in 3′ end. These 3′ cDNA fragments were purified using magnetic bead precipitation and the Illumina adapter 1 (GEX adapter 1) was added to new 5′ end. The junction of Illumina adapter 1 and CATG site was recognized by MmeI, which cut at downstream CATG site and produce fragment of 17 bp tags with adaptor 1. After removing 3′ fragments with magnetic bead precipitation, the Illumina adapter 2 (GEX adapter 2) was ligated to 3′ end of the cDNA tag. These cDNA fragments represented the tag library. After denaturation, the single chain molecules are fixed onto the Illumina sequencing chip (flow cell). Then these sequences were prepared for Solexa sequencing.

### Solexa sequencing

Sequencing was performed by Beijing Genomic institution. PCR phusion amplification with 15 cycles was performed with primers complementary to the adapter sequences to enrich the samples for the desired fragments. The 85 base strips were selected and purified by 6 % TBE PAGE Gel electrophoresis, and then digested and the single-chain molecules were fixed onto the Solexa Sequencing Chip (flow cell). Four color-labeled nucleotides were added, and sequencing was performed with the method of sequencing by synthesis. Image analysis and base-call were performed by the Illumina pipeline, and cDNA sequence tags were revealed after purity filtering. The tags passing initial quality tests were sorted and counted. Each tunnel generates millions of raw reads with sequencing length of 35 bp (target tags plus 3′adaptor). Each molecule in the library represented a single tag derived from a single transcript.

### Analysis and mapping of gene expression (DGE) tags

Clean-tags were obtained by filtering the adaptor sequences and removing low-quality sequences (containing ambiguous bases) and then mapped to the reference genome and genes, which were available at ftp://maizesequence.org/pub/maize/release-5b. Only the tags with perfect match or one mismatch were further considered and annotated based on the reference genes. The expression level of each gene was estimated by the frequency of clean tags and then normalized to TPM (number of transcripts per million clean tags) [1], which is a standard method and extensively used in DGE analysis [43]. The expression level of each gene was measured by the normalized number of matched clean tags, and then KOG functional classification, Gene Ontology (GO) and pathway annotation and enrichment analyses were based on the NCBI COG (http://www.ncbi.nlm.nih.gov/COG) [52], Gene Ontology Database (http://www.geneontology.org/) [23] and KEGG pathway (http://www.genome.jp/kegg/)(Ogata et al. [46],respectively.

### Identification of different expression genes in two libraries

The probability that one gene G is equally expressed in two samples can be illustrated by the following formula:

$$p(x|y) = \left( {\frac{{N_{2} }}{{N_{1} }}} \right)\frac{(x + y)!}{{x!y!\left( {\frac{{N_{2} }}{{N_{1} }}} \right)^{(x + y + 1)} }}\begin{array}{*{20}c} {C(y \le y_{\hbox{min} } |x) = \sum\limits_{y \to 0}^{{y \le y_{\hbox{min} } }} {p(y|x)} } \\ {D(y \ge y_{\hbox{max} } |x) = \sum\limits_{{y \ge y_{\hbox{max} } }}^{\infty } {p(y|x)} } \\ \end{array}$$

N1 and N2 denotes the total number of clean tags in two compared libraries, respectively, while x and y represents the clean tags mapping to gene G. *P* value indicates the significance of prospect differences of transcript accumulation. A combination of FDR < 0.001 and the absolute value of log2-Ratio >= 1 were used as the threshold to determine the significance of gene expression difference in this research.

### GO and pathway enrichment analysis of DEGs

We obtained the GO terms for each maize gene using Blast2GO (version 2.3.5) (http://www.blast2go.org/) with the default parameters. Blast2GO was also used for a GO functional enrichment analysis of certain genes, by performing Fisher’s exact test with a robust FDR correction to obtain an adjusted *P* value between certain test gene groups and the whole genome annotation.

### Validate the DGEs by Real-time PCR

To detect the expression patterns of candidate genes, high-resistance maize inbred line R15 was used in this study, [59] total RNA was isolated form maize leaf sheaths exposed to *R. solani* with 36, 40, 44, 48 h. Control samples were harvested from water-treated leaf sheaths incubated under the same conditions. To validate the DGEs obtained from Solexa sequencing, 12 DGEs were subjected to quantitative real-time PCR analysis using ABI7500. Actin1 (GRMZM2G126010) was used as the endogenous control and cDNA synthesis was carried out using 1 μg total RNA. The corresponding primers were designed by primer5 software and listed in (Support information Table S2) The amplification programs were performed according to the standard protocol of the ABI7500 system: 95 °C for 30 s; 95 °C for 5 s, 60 °C for 30 s, 40 cycles, and followed by a thermal denaturing step to generate the melt curves for verification of amplification specificity. All reactions were run in triplicate, including non-template controls. The threshold cycles (Ct) of each tested genes were averaged for triplicate reactions and the values were normalized according to the Ct of the control products of Actin1 gene. Statistical analysis was performed using the 2^-△△CT^ method.

## Results

### Characterization of the sequenced Solexa libraries

To identify genes involved in maize sheaths infected by *R. solani*, two maize Solexa libraries were constructed from tissues of maize sheaths without any leaves or development leaves, including 4-d and 2-ck libraries. Sequencing depths of 4,200,000 and 4,200,000 tags were achieved in the two libraries respectively, after filtering low quality tags (tags containing ‘*N*’ and adaptor sequences), 4,006,778 and 3,995,176 tags (distinct tags) were remained in 4-d and 2-ck libraries respectively. Considering of the robustness of subsequently data analysis, tags recorded only once were first wiped off owing to their unreliability; leaving 193,222 and 204,824 distinct tags in each library that were detected multiple times (clean tags). In addition, there were 11,602 more unique tags in the 4-d than in the 2-ck library, possibly representing genes related to pathogen interaction and symptom development. The frequency of these tags is shown in (Table 1), which comprises copy numbers in the range 2–100 or higher, of which, the majority of clean tags (62.9 % from each) were present at low copy numbers (<10 copies), and approximately 33.5 % tags from each library were counted between 11 and 100 times. Only approximately 3.6 % tags were detected more than 100 times. The percentage of unique tags rapidly declined as copy number increased, indicating only a small portion of the transcripts was expressed at high level in the conditions tested.

**Table 1:** Basic statistics of tags in treat (4-d) and control sample (2-CK)

	2-ck	4-d
Total tag	4,200,000	4,200,000
Distinct tag	4,006,778	3,995,176
Clean tag (Unique tag copy number >= 1)	193,222	204,824
Unique tag copy number (2-5)	78,699	89,784
Unique tag copy number >5	42,796	44,194
Unique tag copy number >10	30,791	30,924
Unique tag copy number >20	21,548	21,156
Unique tag copy number >50	12,380	11,881
Unique tag copy number >100	7,008	6,885

### Annotation of clean tag

To identify the genes corresponding to the 193,222 and 204,824 meaningful tags in each library, the clean tags were mapped to the reference database [25, 37]. In this study, we used blastn to map the unique tags against the reference genome and gene sequences of maize respectively, an essential dataset containing 32,540 reference genes expressed in the maize genome (http://maizesequence.org/index.html) was prepared by expressed gene analysis. Only the clean tags that matched perfectly or with one mismatch were analyzed further. By assigning the experimental Solexa tags to the virtual reference ones (Table 2), we observed that 159,532(56.7 %) and 110,518 (53.9 %) tags were perfectly matched to the reference genes in 2-ck and 4-d respectively. In addition, approximately 15 % tags in two libraries were mapped to the antisense strands, demonstrating that those regions might be bidirectional transcribed. However, the large proportion of non-matched clean tags revealed that the efficiency of annotation was low when the copy number was between two and five, which is in accordance with the studies of the transcriptomes of zebrafish.

**Table 2:** Summary of Solexa distinct tag-to-gene mapping data

Tag mapping	Distinct tags	
	2-ck	4-d
Sense		
Perfect match	48,260 (25.0 %)	51,191 (25 %)
bp mismatch	25,243 (13.1 %)	23,159 (11.3 %)
Antisense		
Perfect match	29,518 (15.3 %)	29,948 (14.6 %)
1 bp mismatch	6,511 (3.37 %)	6,220 (3.03 %)
All tags mapping to gene	159,532 (56.7 %)	110,518 (53.9 %)
Tags mapping to genome	35,836 (18.6 %)	29,645 (18.5 %)
No matched tags	52,389 (27.1 %)	64,377 (31.4 %)
Total distinct tags (clean tags)	193,222	204,824

**Fig. 1:**
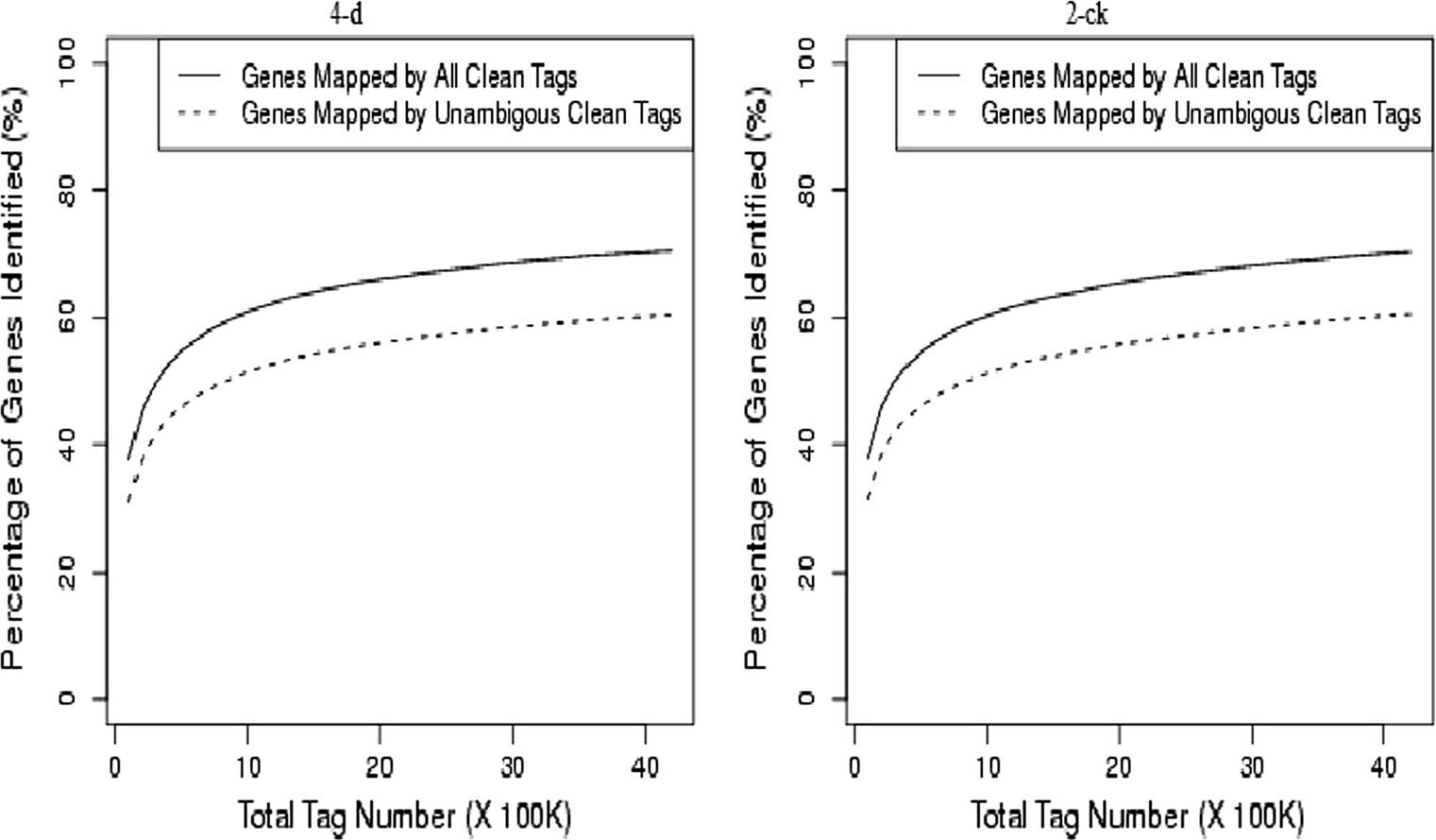
Accumulation the genes mapped by all clean tags (*solid line*) and unique clean tags (*broken line*) in two libraries.B1 to B2 denote 2-ck and 4-d (treat by BLSB), respectively. Percentage of gene identified (*y axis*) increases as the total tag number(*x axis*) increase

It is shown that 4-d and 2-ck libraries were sequenced to saturation in Fig. 1, only fewer tags were identified as the number of sequencing tags increases, producing a full representation of the transcripts in the conditions tested. In conclusion, 4-d library has the relative higher mapping efficiency than 2-CK, indicates that more transcripts have been expressed in development of sheath tissues infected by *R. solani* in 4-d library than in 2-CK.

### Comparison and analysis of differentially expressed genes

**Fig. 2:**
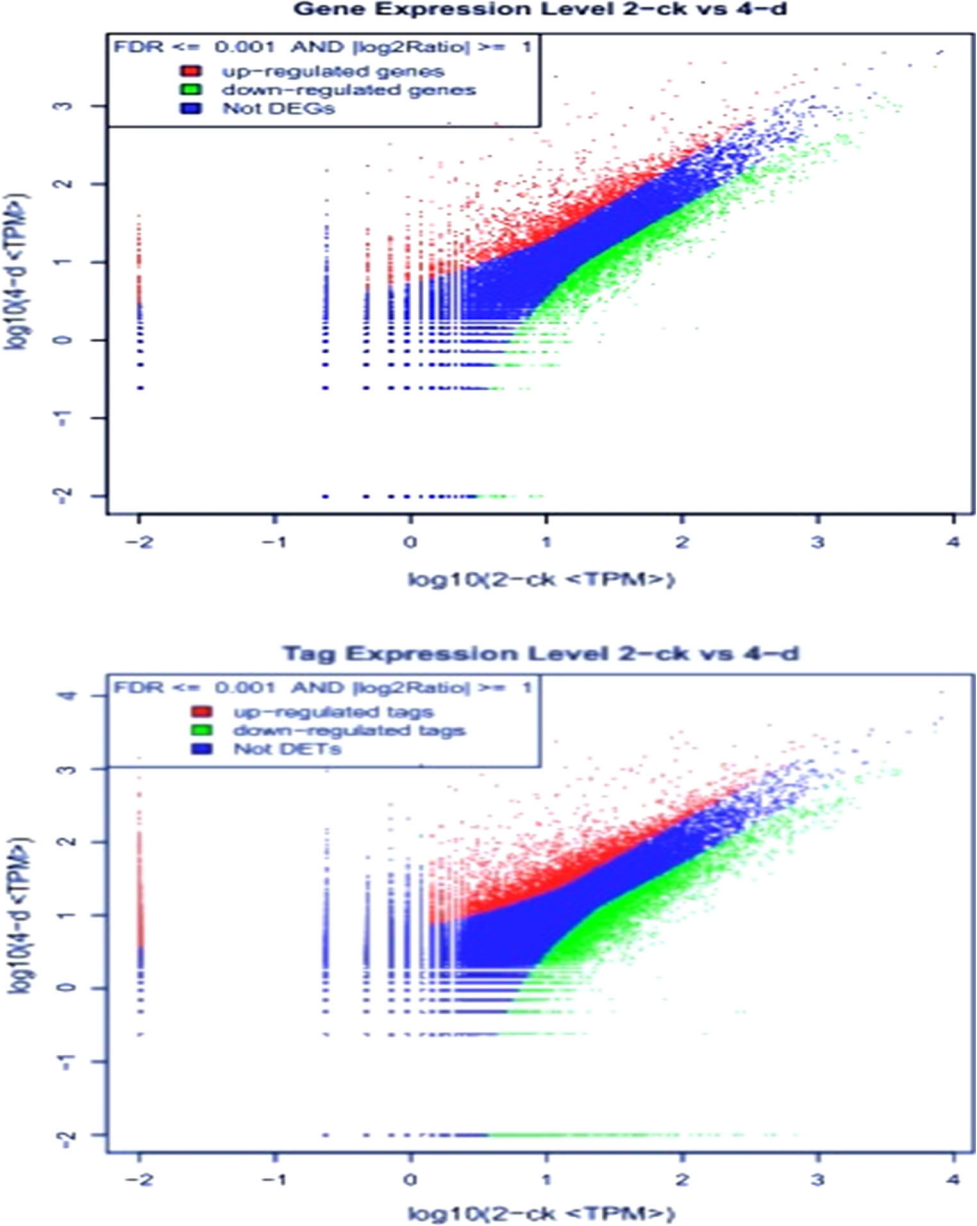
Compare gene expression in two libraries. Gene Expression between 2-ck and 4-d (treat by BLSB) was analyzed, respectively. *Blue dots* represent the transcripts with no significant expression. *Red dots* and *green dots* represent transcripts more abundant in the stage sample and control, respectively. “FDR < 0.001” and “absolute value of log2 Ratio ≥ 1” were used as the thresholds to judge the significance of gene expression difference. (Color figure online)

**Fig. 3:**
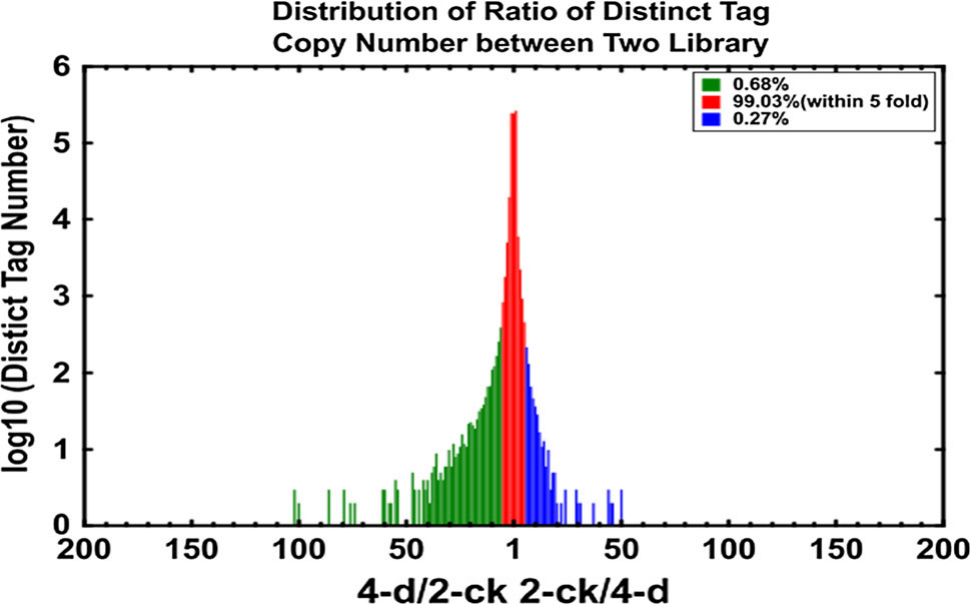
Tags with different expression in stage samples (6-d) compared to control sample (2-ck). Red region represents the differentially expressed tags with differentia expression less than five folds. *Blue* and *green* region represent the up- and down-regulated tags for more than five folds, respectively. (Color figure online)

**Fig. 4:**
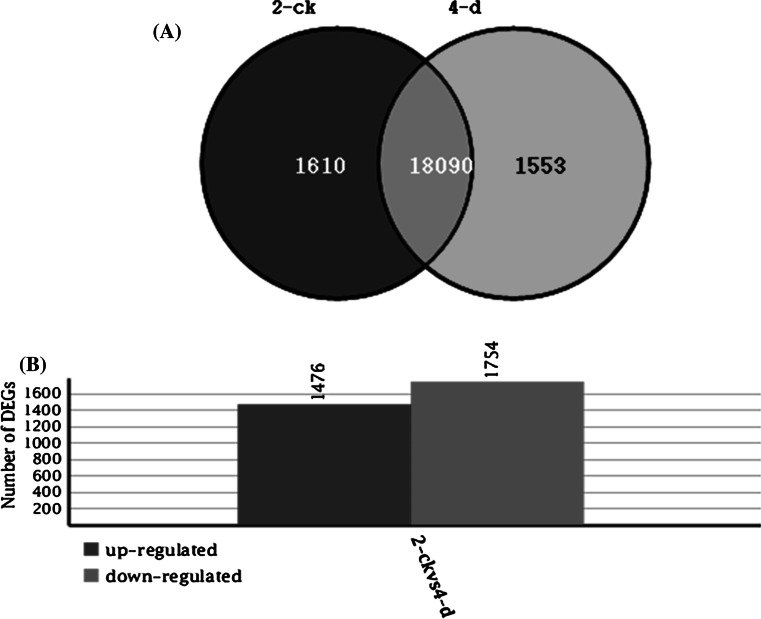
Differentially Expressed Genes in maize between 2-Ck and 4-d (treated by BlSB). Venn diagram (A) and statistical analysis (B) of differentially expressed genes between 2-ck and 4-d

**Fig. 5:**
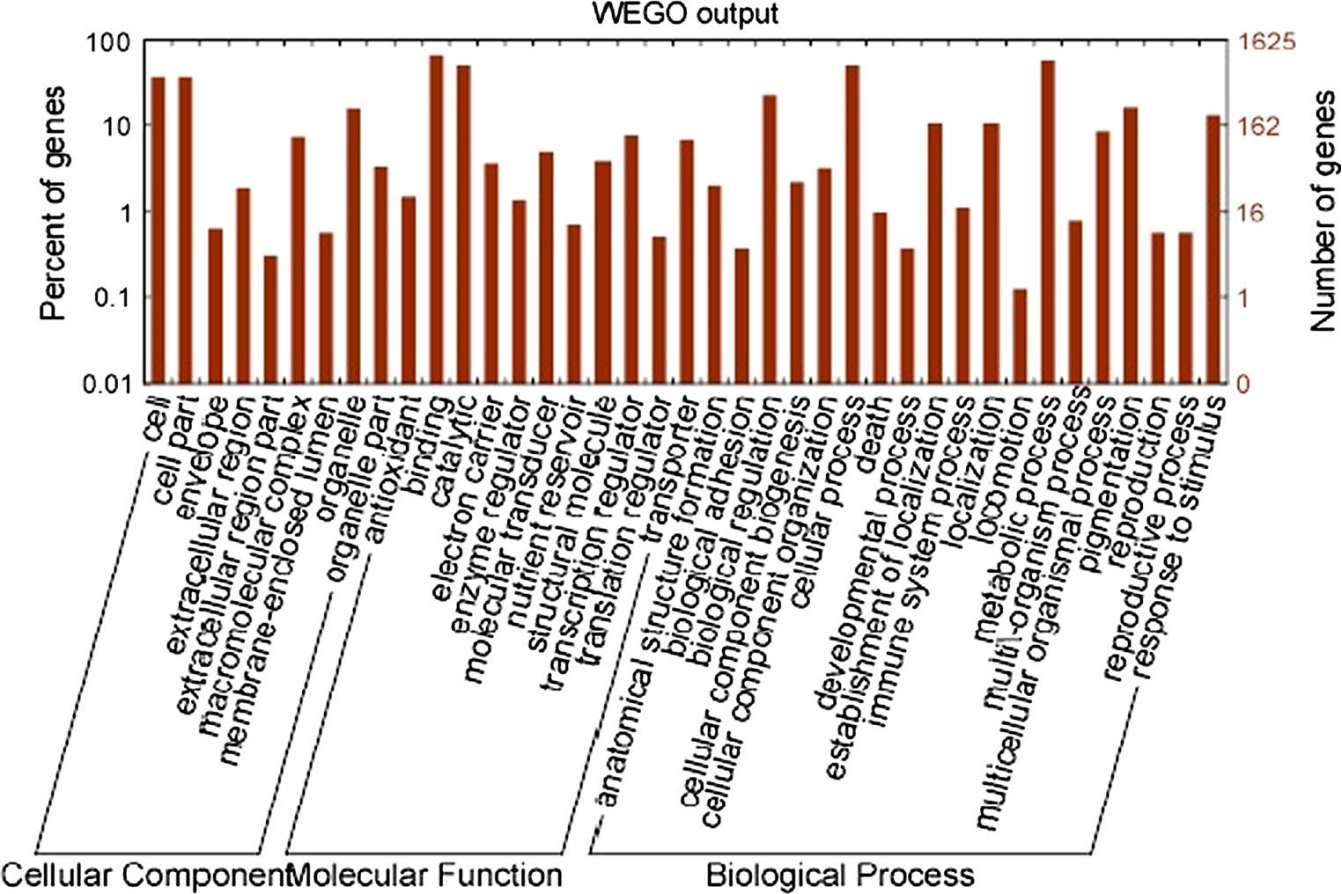
Go annotations of differentially Genes between CK-2 and 4-d

After mapping the tags against the reference genes, the count of the tag corresponding to each gene was calculated in each of the two libraries and used to estimate the gene expression level. The transcripts detected with at least two-fold differences (FDR < 0.001 and absolute values of log2 (Ratio) >= 1) in the 4-d compare with the 2-CK are shown in Fig. 2. The statistic difference of accumulation of unique tags between them was shown in Fig. 3. To study a subset of genes that were associated with maize sheath infected by *R. solani* and to assess the molecular basis of its disease-resistant, we analyzed the most differentially regulated tags with a log2 ratio >2 or <−2 using a greater statistically significant value (*P* < 0.001) as well as false discovery rates (FDR < 0.01), representing 1,476 up-regulated and 1,754 down-regulated transcripts. According to the Venn diagram, there are 1,610 differential genes in 2-ck and 1,553 in 4-d respectively in Fig. 4. Apart from the unknown transcripts (55 %), predicted or known genes were categorized according to their functions. GO functional annotation of DEGs indicated that both up-regulated genes and down-regulated ones can be classified into 11 categories in Fig. 5, such as catalytic, electrical carrier, transcription regulation and enzyme regulator, and so forth. Significant GO analysis of DEGs in molecular function showed that the top one was transferase activity, transferring hexosyl groups, it is interesting that most of DEGs involved in biological process such as response to stimulus, multicellular organismal process and response to abiotic stimulus in (Support information Table 3). Among these highly expressed genes, some of which were associated with defense, transport, transcription, signal transduction and metabolism and the others were associated with senescence, abiotic and biotic stresses. (Support information Table S1).

### Pathway enrichment analysis of DEGs

The *R. sonali* affected biological pathways were evaluated by enrichment analysis of DEGs. Significantly enriched metabolic pathways and signal transduction pathways were identified. A total of 96 and 100 pathways were affected by up-regulated DEGs and down-regulated DEGs, respectively (Support information Table 5), in addition to 11 KO numbers with different expression genes between 4-d and 2-ck were not contained in KEGG (Support information Table 6), most of them were found in the enriched pathways and the first twenty enriched pathways were reported in (Support information Table 4). Pathways with Q value <0.05 are considerably enriched. Caffeine metabolism proteins constituted the only significantly affected pathway for the upregulated DEGs (Q < 0.05). Other non-significant enriched pathways with a large number of unregulated DEGs included betalain biosynthesis and Nicotinate and nicotinamide, ether lipid metabolism, non-homologous end-joining and proteasome. Moreover, there were more significantly enriched pathways for the down-regulated DEGs, which were involved in photosynthesis, as well as metabolism of vitamin B6, sphingolipid, arachidonic acid and degradation of glycosaminoglycan, along with biosynthesis of N-glycan, carotenoid and fatty acid elongation in mitochondria.

### Quantitative real-time PCR (qRT-PCR) confirmation

**Fig. 6:**
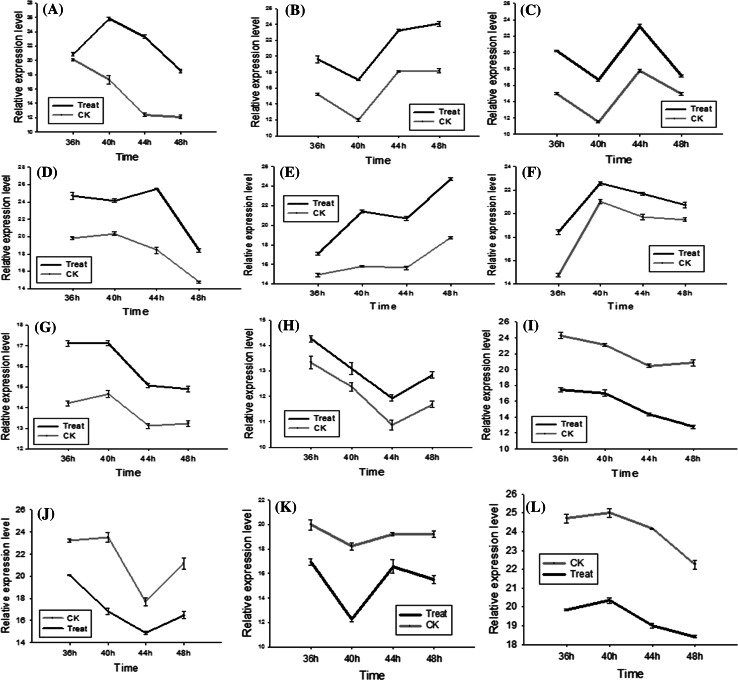
The 12 candidate DGEs validated by Q-PCR technology. Note: A-L represented the DGEs were as follows respectively. A: GRMZM2G397755, B: GRMZM2G151425, C: GRMZM2G332390, D: GRMZM2G012242, E: GRMZM2G113860, F: GRMZM2G136372, G: GRMZM2G123119, H: GRMZM2G179896, I: GRMZM2G065203, J: GRMZM2G085924,K: GRMZM2G059191, L: GRMZM2G303419.Their function and expression levels in DGE library were shown in Table S2

To evaluate the validity the DGEs obtained from Solexa sequencing and to further assess the patterns of differential gene expression, 12 candidate genes were selected and detected by qRT-PCR, including 8 up-regulated DGEs and 4 down-regulated DGEs (Support information Table S2). As shown in Fig. 6, the expression patterns showed general agreement with the Solexa sequencing, but the apparent discrepancies with respect to ratio was clearly found, transcripts from highly abundant Solexa tags appeared at the expected lower cycle numbers in the quantitative PCR analyses. It should be attributed to the essentially different algorithms determined by the two techniques (DEG and Q-PCR).

## Discussion

The major goal of the present study is to preliminarily explore disease-resistant transcripts involved in the maize sheath infected by *R. solani*, as well as to provide foundation for investigating their regulation mechanisms. To our knowledge, this is the first report that comprehensively shows the transcriptional changes during the development of maize sheath inoculated with *R. solani*. We used the Illumina/Solexa DGE system, which is essentially a serial analysis of gene expression-based tag profiling approach. The results provided estimates of gene expression as determined by the frequency that any given tag (representing a transcript) is sequenced (Fig. 2). The data indicates that there is sufficient coverage depth to reach saturation, that is, a complete assessment of all transcripts present in the libraries. In addition, expression levels of candidate genes were confirmed by Q-PCR technology. Although the differences in gene expression did not match the magnitude of those detected by Solexa-based sequencing method, the trends of up- and down- regulation were similar, it may be owe to the apparent discrepancies with respect to ratio and the essentially different algorithms determined by the two techniques. In conclusion, Solexa sequencing has been documented to be more sensitive for estimation of gene expression, especially for low-abundance transcripts compared to microarrays and Real-time RT-PCR [1].

### Functional analysis of DEGs

A set of transcripts was clearly more abundant in leaf sheaths after *R. solani* infection in 4-d compared to 2-CK. This group possibly contains elements that confer resistance to the spread of the pathogen in “R15”. These transcripts likely encode genes responding to the pathogen that underlie genetic resistance, which were broadly grouped into the following categories based on their known roles combing GO enrichment analysis with scientific literature.

### Defense response genes

Among defense response genes, in addition to thaumatin-like protein [47], plant disease resistance response protein, Flagellar hook-length control protein, and harpin-induced protein-related [16, 28] were found in our research, major facilitator superfamily, Mlo-related protein, pathogenesis-related transcriptional factor, pectinesterase inhibitor and pistil-specific extensin-like protein were also enriched in this study which have been widely studied in plant pathogen resistance. Moreover, a new transcript, universal stress protein A was found in this study, which has been best characterized and found to be highly expressed in response to heat, substrate starvation, exposure to antimicrobial agents and oxidative stress in *Escherichia coli* K-12. In our research, Universal stress protein A (+11.03 fold in 4-d vs 2-ck) showed the great trend in 4-d than 2-CK and was found associated with *R. solani* attack and may also play an important role in respond to *R. solani* attack.

### Transport

Several transcripts associated with transport function were found in this research. Multi antimicrobial extrusion protein MatE (+4.58 fold in 4-d vs 2-d) and ABC transporter (+5.58 fold in 4-d vs 2-CK) are well known transporters in clinical study for bacterial infection of human [41]. In plant, plant ABC transporters have been demonstrated to participate in chlorophyll biosynthesis, formation of Fe/S clusters, stomatal movement, and probably ion fluxes; hence they may play a central role in plant growth and developmental processes. And plant mates have been genetically identified, characterized and found to be involved in the detoxification of endogenous secondary metabolites and xenobiotics. Another gene identified to be transport related is mitochondrial carrier protein (+11.17 fold in 4-d vs 2-CK) which might be involved in the excretion of organic acids and rhizotoxic aluminum tolerance [15]. Moreover, sugar transporter and ATPase, P-type, K/Mg/Cd/Cu/Zn/Na/Ca/Na/H-transporter were also demonstrated in our study, however, there was only a little report about these transporters, which might play a purely nutritional role and supply sugars and K/Mg/Cd/Cu/Zn/Na/Ca/Na/H to cells for growth and development, whereas others are involved in generating osmotic gradients required to drive mass flow or movement.

### Signal transduction

There were 36 transcripts in our results associated with signal transduction. Four came from genes (GRMZM2G048131, GRMZM2G074742, GRMZM2G121309, GRMZM2G163848) encoding AUX/IAA proteins that were more prevalent (4.5–6.2 fold) in the 4-d library than in 2-CK. It is reported that exogenous salicylic acid (SA) treatment triggers the stabilization of AUX-IAA (auxin/indole-3-acetic acid) proteins that negatively regulate auxin signaling, thus attenuating auxin signaling [58], probably indirectly through repression of the F-box auxin receptor TIR1, thereby decreasing sensitivity to auxin treatment and limiting ubiquitination of AXR2 and other AUX-IAA proteins. Various signals presented in our results, including auxin, abscisic acid (ABA), as well as intracellular messengers like calcium has been proposed to regulate plant responses in adverse environmental conditions and thus contribute to the coordination of plant stress physiology [63]. Activation of ABA biosynthetic and signaling pathways promotes disease susceptibility to several plant pathogens [5]; Ton and Mauch-Mani [53], and most of RING-H2 genes were annotated in this study which has demonstrated regulatory function in ABA signaling, drought tolerance, regulation of growth and defense responses against abiotic/biotic stresses [30, 38]. Moreover, six transcripts (GRMZM2G041729, GRMZM2G419452, GRMZM2G040094, GRMZM2G366411, GRMZM2G001814, and GRMZM2G021998) were associated with calcium signaling pathway. All of these are also induced by senescence and stresses [18, 34].

### Transcription

49 transcripts associated with tyrosine protein kinase transcription were 4–8.033 fold more abundant in 4-d than 2-CK libraries. Transcripts annotated as zinc-finger protein 1, Transcriptional factor B3, AP2 domain class transcription factor, basic helix-loop-helix protein, DNA-binding WRKY, Calcium-binding EF-hand, Leucine-rich repeat protein were all present at higher steady state levels in infected tissue. They have been documented to play important roles in responding to phytohormone stasis, pathogen attack and environmental stresses [17, 19, 20, 51] and all of which have been observed to increase in response to pathogen challenge [50]. And also these defense-associated TFs can regulate downstream defense-related genes, and may be regulated by phosphorylation themselves [31].

## Metabolism

### Protein metabolism

Several transcripts related to protein metabolism were found abundant in 4-d library, Among them, protein phosphatase 2c (4.58–5.58 fold in 4-d VS 2-CK) regulates numerous ABA responses [22, 57]. Recent studies place protein phosphatases in various signaling cascades including those for ABA, pathogen and stress responses, and developmental processes. It is clearly demonstrated that protein phosphatases function not only by counterbalancing the protein kinases but also by taking a leading role in many signaling events [40]. Protein kinase (4.58–6.57 fold in 4-d VS 2-CK) play a central role in signaling during pathogen recognition and the subsequent activation of plant defence mechanisms [48]. In addition, phosphate-induced protein 1(GRMZM2G119766) has been shown to be involved in MAP kinase pathways. In plants, there is evidence for MAPKs playing a role in the signaling of abiotic stresses, pathogens and plant hormones.

### Secondary metabolism

This subcategory contained four genes, including a higher level of polyketide synthase, (4.48–5.58 fold in 4-d vs CK) in infected tissues, it is consistent with previous reports that it will be more abundant after pathogen infection and the secondary metabolite synthesis depends on polyketide synthase[6]. Isopenicillin N synthase (GRMZM2G031432, GRMZM2G0317241, 5.58 and 6.89 fold in 4-d vs CK) has also played a potential pathogen resistance role because it is involved in biosynthesis of Peroxisomes, an important product in resistance to pathogen infection[6]. UDP-glucose glucosyltransferase (+ 4.28–7.82 fold in 4-d vs 2-CK) are reported to be induced by abiotic stresses [21, 45].

In addition, other highly expressed metabolic genes in the 4-d samples were plant lipid transfer protein, cytochrome P450 and thioredoxin-like. It is reported that the role of LTP in the physiology of plants, including plant defense against phytopathogens [9, 55, 65]. Cytochrome P450 and thioredoxin-like are related to carbohydrate metabolism, photosynthesis and thioredoxin synthesis. Thioredoxins are ubiquitous disulfide reductases that regulate the redox status of target proteins which was involved in oxidative damage avoidance by supplying reducing power to reductases detoxifying lipid hydroperoxides or repairing oxidized proteins and could act as regulators of scavenging mechanisms and as components of signalling pathways in the plant antioxidant network [56].

### Transcripts less abundant in infected sheaths

The most striking functions for transcripts less abundant in infected sheath were those associated with carotenoid biosynthesis. DEGs were detected to be less prevalent in the 4-d libraries more than fourfold compared to CK, most of which, such as O-methyltransferase, COMT [24], ferredoxin–NADP reductase [10], phospholipid/glycerol acyltransferase [11], S-adenosyl-l-Met (SAM) dependent carboxyl methyltransferase [33].Of which, O-methyltransferase, COMT is reported to be positively associated with plant defense responses to pathogen attack. However, our data indicated that the expression level of these transcripts was lower in infected sheath (Fig. 6).

Ferredoxin-NADP+(oxido)reductase (EC 1.18.1.2, FNR) is an FAD-containing enzyme that catalyzes the reversible electron transfer between NADP(H) and electron carrier proteins such as ferredoxin and flavodoxin [3]. Of which, ferredoxin (Fd) is a fundamental protein that is involved in several metabolic pathways. The amount of Fd found in plants is generally regulated by environmental stress, including biotic and abiotic events and Fd levels were increased by inoculation with Pseudomonas syringae pv. syringae but were reduced by *Erwinia carotovora* ssp. Carotovora [26], but we found Fd levels were reduced by BLSB through reducing Ferredoxin-NADP+(oxido)reductase in this research. In addition, it is reported that the acyl-CoA-independent synthesis of TAG is mediated by phospholipid:diacylglycerol acyltransferase in plants and it is proposed to be involved in the accumulation of high levels of hydroxylated fatty acid (ricinoleic acid) and epoxidated fatty acid (vernolic acid) [14], these phospholipid-derived molecules are emerging as novel second messengers in plant defence signalling. They may be regulate ion channels and proton pumps by free fatty acids, and then conversion free fatty acids into bioactive octadecanoids such as jasmonic acid to response to pathogen attack. Moreover, SAM dependent carboxyl methyltransferase, the first characterized member of the family, catalyzes the formation of methyl salicylate (MeSA) from SA and SAM [44, 49], and MeSA is component of the floral scents of some plants and are also produced by vegetative parts of plants in response to environmental challenge [12].

### Pathway enrichment analysis of DEGs

Pathway enrichment analysis revealed the most significantly affected pathways during the *R. solani* infection in“R15”. It is not surprising that the “metabolic pathways” was the most affected for the DEGs in 4-d library. This finding implies that the maize resistance material R15 utilizes biosynthesis of plant hormones, terpenoids and steroids, N-glycan, Zeatin to protect itself from the pathogen attack. The second affected pathway was the “Plant-pathogen interaction” pathway. In this pathway genes encoding WRKY family were more prevalent in the 4-d than the 2-ck library. In addition, genes required for plant-pathogen interaction were also affected, such as CDPK, NOS, and JAZ. These genes are involved in the plant-pathogen interaction system, and may regulate plant defense patterns in response to pathogen, thereby linking signaling with metabolism. The other noticeable pathways with a large amount of DEGs associated with BLSB infection were starch and sucrose metabolism, Ubiquitin mediated proteolysis, plant hormone biosynthesis, and splicesome-associated proteins. For DEGs less prevalent in infected versus control libraries, there was significant enrichment for transcripts associated with photosynthesis.

## Conclusions

Solexa-based sequencing can be used for analyzing variation in gene expression between two samples. The gene expression level in high-resistance maize inbred line “R15” sheath infection with *R. solani* (4-d) changed significantly in comparison with control (2-ck). Analysis of differential expressed genes involved in response to pathogen infection allows delineation of candidate genes potentially relevant to *R. solani* resistance in maize. In addition, Our analysis of the transcription and gene expression in “R15” infection by *R. solani* revealed changes in multiple signaling pathways involved in immunity in “R15”, the candidate immune-related genes and association signaling pathways involved in pathogen infection were identified and thereby provided valuable leads for further investigation the immune response in maize.

## Electronic supplementary material

Below is the link to the electronic supplementary material.

Supplementary material 1 (DOCX 1106 kb)
