# Supplementary material for: Genome expression profile analysis of the maize sheath in response to inoculation to R. solani
Source: Mol Biol Rep. 2014 Jan 14;41(4):2471–83. doi: 10.1007/s11033-014-3103-z (PMC3968446; doi:10.1007/s11033-014-3103-z)
Supplement: Supplementary file 1 — Supplementary material 1 (DOCX 1106 kb) [file 11033_2014_3103_MOESM1_ESM.docx]

**Supporting information**

**Figure S1.The culture of AG1-AI (a type of Rhizoctonia solani Kühn) in culture medium**


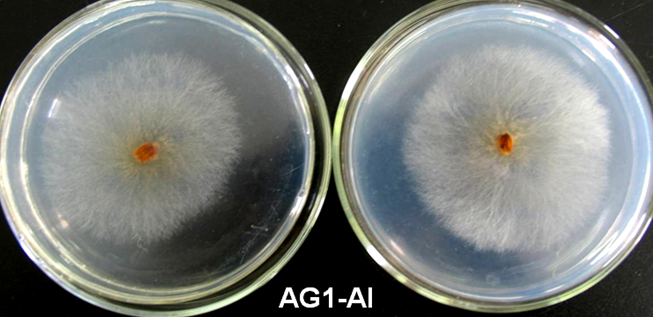


**Figure S2 Observation of maize sheaths infection by R. solani under scanning electron microscope**


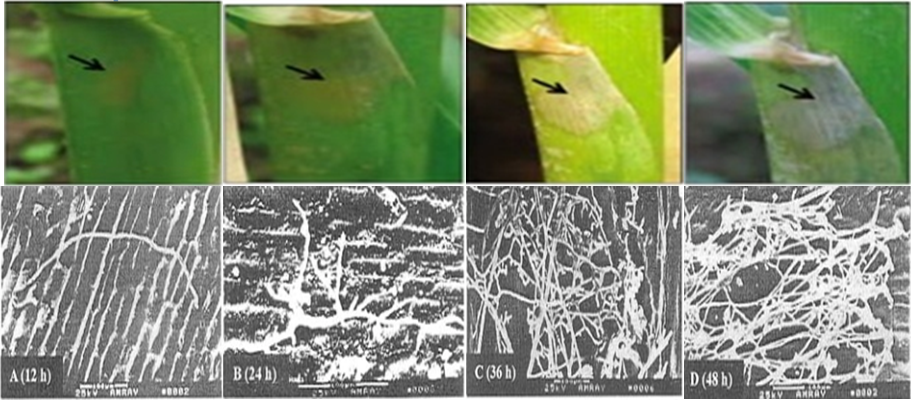


(A) Hyphae growth extends in the sheath cell surface and generates a few branches

(B) Hyphae growth presents a multi branch and staggered mesh structure

(C) Hyphae interweaves to branch and forms the rudiment of infection pad

(D) Hyphae forms a large number of mature infection cushion and begins to infected tissue growth

**Table legends**

**Table 3 Significant Go terms of DEGs in the annotation analysis of biological process and molecular function**

| GO term | Ontology | Description | Number in input list | p-value | FDR |
| --- | --- | --- | --- | --- | --- |
| GO:0050896 | P | response to stimulus | 207 | 3.3E-07 | 0.00022 |
| GO:0032501 | P | multicellular organismal process | 133 | 1.8E-07 | 0.00022 |
| GO:0009628 | P | response to abiotic stimulus | 125 | 3.3E-07 | 0.00022 |
| GO:0009409 | P | response to cold | 117 | 1.3E-06 | 0.00026 |
| GO:0048871 | P | multicellular organismal homeostasis | 117 | 1.3E-06 | 0.00026 |
| GO:0050826 | P | response to freezing | 117 | 1.3E-06 | 0.00026 |
| GO:0009266 | P | response to temperature stimulus | 118 | 9.2E-07 | 0.00026 |
| GO:0006950 | P | response to stress | 185 | 1.2E-06 | 0.00026 |
| GO:0042309 | P | homoiothermy | 117 | 1.3E-06 | 0.00026 |
| GO:0001659 | P | temperature homeostasis | 117 | 1.3E-06 | 0.00026 |
| GO:0065007 | P | biological regulation | 340 | 4.3E-06 | 0.00081 |
| GO:0042592 | P | homeostatic process | 129 | 5.6E-06 | 0.00096 |
| GO:0080090 | P | regulation of primary metabolic process | 205 | 0.000013 | 0.0014 |
| GO:0065008 | P | regulation of biological quality | 132 | 0.000012 | 0.0014 |
| GO:0009889 | P | regulation of biosynthetic process | 195 | 0.000013 | 0.0014 |
| GO:0031326 | P | regulation of cellular biosynthetic process | 195 | 0.000013 | 0.0014 |
| GO:0010556 | P | regulation of macromolecule biosynthetic process | 195 | 0.000013 | 0.0014 |
| GO:0019219 | P | regulation of nucleobase, nucleoside, nucleotide and nucleic acid metabolic process | 194 | 0.000013 | 0.0014 |
| GO:0051171 | P | regulation of nitrogen compound metabolic process | 194 | 0.000013 | 0.0014 |
| GO:0045449 | P | regulation of transcription | 192 | 0.000018 | 0.0019 |
| GO:0031323 | P | regulation of cellular metabolic process | 196 | 0.00002 | 0.002 |
| GO:0060255 | P | regulation of macromolecule metabolic process | 205 | 0.000024 | 0.0022 |
| GO:0010468 | P | regulation of gene expression | 194 | 0.000027 | 0.0025 |
| GO:0019222 | P | regulation of metabolic process | 205 | 0.00004 | 0.0034 |
| GO:0051252 | P | regulation of RNA metabolic process | 132 | 0.00005 | 0.0041 |
| GO:0006629 | P | lipid metabolic process | 72 | 0.00007 | 0.0053 |
| GO:0006355 | P | regulation of transcription, DNA-dependent | 131 | 0.000069 | 0.0053 |
| GO:0032774 | P | RNA biosynthetic process | 135 | 0.00026 | 0.019 |
| GO:0006351 | P | transcription, DNA-dependent | 135 | 0.00025 | 0.019 |
| GO:0006566 | P | threonine metabolic process | 6 | 0.00046 | 0.031 |
| GO:0006350 | P | transcription | 198 | 0.00062 | 0.042 |
| GO:0016758 | F | transferase activity, transferring hexosyl groups | 50 | 2.4E-07 | 0.00033 |
| GO:0050825 | F | ice binding | 117 | 1.3E-06 | 0.00059 |
| GO:0050824 | F | water binding | 117 | 1.3E-06 | 0.00059 |

**Table 4.List of 20 top pathways association with significant Go terms for DEGs according the increasing number and increasing fold**

| Pathway | **Sample1 (1639)** | **Sample2 (14375)** | **Increase NO** | **Increase fold** | **Pvalue** | **Qvalue** | **Pathway ID** |
| --- | --- | --- | --- | --- | --- | --- | --- |
| [Metabolic pathways](file:///C:\Users\gaojian\Desktop\A-MEXP-1701.adf.xls#RANGE!gene4) | 445 | 3173 | 2728 | 7.130337079 | 1.47E-07 | 4.31E-06 | ko01100 |
| [Plant-pathogen interaction](file:///C:\Users\gaojian\Desktop\A-MEXP-1701.adf.xls#RANGE!gene110) | 121 | 1246 | 1125 | 10.29752066 | 0.9795313 | 1.00E+00 | ko04626 |
| [Biosynthesis of phenylpropanoids](file:///C:\Users\gaojian\Desktop\A-MEXP-1701.adf.xls#RANGE!gene7) | 126 | 762 | 636 | 6.047619048 | 8.40E-06 | 1.40E-04 | ko01061 |
| [Biosynthesis of plant hormones](file:///C:\Users\gaojian\Desktop\A-MEXP-1701.adf.xls#RANGE!gene6) | 110 | 643 | 533 | 5.845454545 | 6.88E-06 | 1.34E-04 | ko01070 |
| [Ribosome](file:///C:\Users\gaojian\Desktop\A-MEXP-1701.adf.xls#RANGE!gene12) | 92 | 557 | 465 | 6.054347826 | 0.0001451 | 1.41E-03 | ko03010 |
| [Spliceosome](file:///C:\Users\gaojian\Desktop\A-MEXP-1701.adf.xls#RANGE!gene117) | 32 | 462 | 430 | 14.4375 | 0.9995953 | 1.00E+00 | ko03040 |
| [Phenylpropanoid biosynthesis](file:///C:\Users\gaojian\Desktop\A-MEXP-1701.adf.xls#RANGE!gene13) | 84 | 502 | 418 | 5.976190476 | 0.00018001 | 1.62E-03 | ko00940 |
| [Starch and sucrose metabolism](file:///C:\Users\gaojian\Desktop\A-MEXP-1701.adf.xls#RANGE!gene61) | 51 | 419 | 368 | 8.215686275 | 0.329703 | 6.32E-01 | ko00500 |
| [Biosynthesis of terpenoids and steroids](file:///C:\Users\gaojian\Desktop\A-MEXP-1701.adf.xls#RANGE!gene27) | 61 | 407 | 346 | 6.672131148 | 0.01525128 | 6.61E-02 | ko01062 |
| [Ubiquitin mediated proteolysis](file:///C:\Users\gaojian\Desktop\A-MEXP-1701.adf.xls#RANGE!gene105) | 27 | 319 | 292 | 11.81481481 | 0.9650319 | 1.00E+00 | ko04120 |
| [Biosynthesis of alkaloids derived from terpenoid and polyketide](file:///C:\Users\gaojian\Desktop\A-MEXP-1701.adf.xls#RANGE!gene17) | 54 | 307 | 253 | 5.685185185 | 0.00075898 | 5.22E-03 | ko01066 |
| [Oxidative phosphorylation](file:///C:\Users\gaojian\Desktop\A-MEXP-1701.adf.xls#RANGE!gene100) | 25 | 275 | 250 | 11 | 0.9087992 | 1.00E+00 | ko00190 |
| [Biosynthesis of alkaloids derived from histidine and purine](file:///C:\Users\gaojian\Desktop\A-MEXP-1701.adf.xls#RANGE!gene25) | 44 | 272 | 228 | 6.181818182 | 0.01046516 | 4.73E-02 | ko01065 |
| [Stilbenoid, diarylheptanoid and gingerol biosynthesis](file:///C:\Users\gaojian\Desktop\A-MEXP-1701.adf.xls#RANGE!gene20) | 47 | 270 | 223 | 5.744680851 | 0.0020044 | 1.17E-02 | ko00945 |
| [Pyrimidine metabolism](file:///C:\Users\gaojian\Desktop\A-MEXP-1701.adf.xls#RANGE!gene95) | 22 | 237 | 215 | 10.77272727 | 0.8744072 | 1.00E+00 | ko00240 |
| [Purine metabolism](file:///C:\Users\gaojian\Desktop\A-MEXP-1701.adf.xls#RANGE!gene46) | 35 | 247 | 212 | 7.057142857 | 0.1026934 | 2.56E-01 | ko00230 |
| [Endocytosis](file:///C:\Users\gaojian\Desktop\A-MEXP-1701.adf.xls#RANGE!gene114) | 11 | 203 | 192 | 18.45454545 | 0.9989555 | 1.00E+00 | ko04144 |
| [Cysteine and methionine metabolism](file:///C:\Users\gaojian\Desktop\A-MEXP-1701.adf.xls#RANGE!gene51) | 28 | 206 | 178 | 7.357142857 | 0.186061 | 4.27E-01 | ko00270 |
| [Peroxisome](file:///C:\Users\gaojian\Desktop\A-MEXP-1701.adf.xls#RANGE!gene87) | 19 | 192 | 173 | 10.10526316 | 0.7775171 | 1.00E+00 | ko04146 |
| [Phenylalanine metabolism](file:///C:\Users\gaojian\Desktop\A-MEXP-1701.adf.xls#RANGE!gene29) | 33 | 199 | 166 | 6.03030303 | 0.01726432 | 6.97E-02 | ko00360 |

**Table 5.list of identity of genes with different expression and the corresponding KO numbers between 4-d and 2-ck in KEGG**

| #Pathway | Sample1 (1639) | Sample2 (14375) | Pvalue | Qvalue | Pathway ID | Gene classification |
| --- | --- | --- | --- | --- | --- | --- |
| Photosynthesis | 44 | 124 | 1.51E-12 | 1.61E-10 | ko00195 | Down-regulated |
| [Photosynthesis - antenna proteins](DGE---Gaojian/纹枯病DGE/upload/pathway/2-ckvs4-d.htm#gene2) | 19 | 28 | 2.75E-12 | 1.61E-10 | ko00196 | Down-regulated |
| [Carbon fixation in photosynthetic organisms](DGE---Gaojian/纹枯病DGE/upload/pathway/2-ckvs4-d.htm#gene3) | 41 | 128 | 3.38E-10 | 1.32E-08 | ko00710 | Both |
| [Pentose phosphate pathway](DGE---Gaojian/纹枯病DGE/upload/pathway/2-ckvs4-d.htm#gene5) | 21 | 64 | 4.42E-06 | 1.03E-04 | ko00030 | Both |
| [Glycolysis / Gluconeogenesis](DGE---Gaojian/纹枯病DGE/upload/pathway/2-ckvs4-d.htm#gene8) | 43 | 199 | 2.42E-05 | 3.54E-04 | ko00010 | Both |
| [Glutathione metabolism](DGE---Gaojian/纹枯病DGE/upload/pathway/2-ckvs4-d.htm#gene9) | 31 | 131 | 5.37E-05 | 6.98E-04 | ko00480 | Both |
| [Ribosome](DGE---Gaojian/纹枯病DGE/upload/pathway/2-ckvs4-d.htm#gene12) | 92 | 557 | 0.0001451 | 1.41E-03 | ko03010 | Both |
| [Phenylpropanoid biosynthesis](DGE---Gaojian/纹枯病DGE/upload/pathway/2-ckvs4-d.htm#gene13) | 84 | 502 | 0.00018 | 1.62E-03 | ko00940 | Both |
| [Amino sugar and nucleotide sugar metabolism](DGE---Gaojian/纹枯病DGE/upload/pathway/2-ckvs4-d.htm#gene14) | 36 | 178 | 0.0004224 | 3.53E-03 | ko00520 | Both |
| [Metabolism of xenobiotics by cytochrome P450](DGE---Gaojian/纹枯病DGE/upload/pathway/2-ckvs4-d.htm#gene15) | 25 | 111 | 0.0006062 | 4.43E-03 | ko00980 | Both |
| [Fructose and mannose metabolism](DGE---Gaojian/纹枯病DGE/upload/pathway/2-ckvs4-d.htm#gene16) | 25 | 111 | 0.0006062 | 4.43E-03 | ko00051 | Both |
| [Glycine, serine and threonine metabolism](DGE---Gaojian/纹枯病DGE/upload/pathway/2-ckvs4-d.htm#gene18) | 21 | 92 | 0.0013388 | 8.70E-03 | ko00260 | Both |
| [Flavonoid biosynthesis](DGE---Gaojian/纹枯病DGE/upload/pathway/2-ckvs4-d.htm#gene19) | 46 | 259 | 0.0014675 | 9.04E-03 | ko00941 | Both |
| [Stilbenoid, diarylheptanoid and gingerol biosynthesis](DGE---Gaojian/纹枯病DGE/upload/pathway/2-ckvs4-d.htm#gene20) | 47 | 270 | 0.0020044 | 1.17E-02 | ko00945 | Both |
| [Porphyrin and chlorophyll metabolism](DGE---Gaojian/纹枯病DGE/upload/pathway/2-ckvs4-d.htm#gene21) | 14 | 54 | 0.0023502 | 1.31E-02 | ko00860 | Both |
| [Flavone and flavonol biosynthesis](DGE---Gaojian/纹枯病DGE/upload/pathway/2-ckvs4-d.htm#gene22) | 18 | 78 | 0.0025223 | 1.34E-02 | ko00944 | Both |
| [Pyruvate metabolism](DGE---Gaojian/纹枯病DGE/upload/pathway/2-ckvs4-d.htm#gene23) | 24 | 119 | 0.0037308 | 1.90E-02 | ko00620 | Both |
| [Benzoxazinoid biosynthesis](DGE---Gaojian/纹枯病DGE/upload/pathway/2-ckvs4-d.htm#gene24) | 19 | 92 | 0.007116 | 3.47E-02 | ko00402 | Both |
| [Nitrogen metabolism](DGE---Gaojian/纹枯病DGE/upload/pathway/2-ckvs4-d.htm#gene26) | 20 | 102 | 0.0105006 | 4.73E-02 | ko00910 | Both |
| [Terpenoid backbone biosynthesis](DGE---Gaojian/纹枯病DGE/upload/pathway/2-ckvs4-d.htm#gene28) | 15 | 73 | 0.016412 | 6.86E-02 | ko00900 | Both |
| [Phenylalanine metabolism](DGE---Gaojian/纹枯病DGE/upload/pathway/2-ckvs4-d.htm#gene29) | 33 | 199 | 0.0172643 | 6.97E-02 | ko00360 | Both |
| [DNA replication](DGE---Gaojian/纹枯病DGE/upload/pathway/2-ckvs4-d.htm#gene30) | 19 | 102 | 0.0208444 | 8.13E-02 | ko03030 | Both |
| [Regulation of autophagy](DGE---Gaojian/纹枯病DGE/upload/pathway/2-ckvs4-d.htm#gene31) | 14 | 70 | 0.0249291 | 9.41E-02 | ko04140 | Both |
| [Phenylalanine, tyrosine and tryptophan biosynthesis](DGE---Gaojian/纹枯病DGE/upload/pathway/2-ckvs4-d.htm#gene32) | 14 | 71 | 0.0279057 | 9.89E-02 | ko00400 | Both |
| [Tyrosine metabolism](DGE---Gaojian/纹枯病DGE/upload/pathway/2-ckvs4-d.htm#gene33) | 14 | 71 | 0.0279057 | 9.89E-02 | ko00350 | Both |
| [Tropane, piperidine and pyridine alkaloid biosynthesis](DGE---Gaojian/纹枯病DGE/upload/pathway/2-ckvs4-d.htm#gene34) | 8 | 36 | 0.0463152 | 1.59E-01 | ko00960 | Both |
| [beta-Alanine metabolism](DGE---Gaojian/纹枯病DGE/upload/pathway/2-ckvs4-d.htm#gene35) | 11 | 56 | 0.0490612 | 1.64E-01 | ko00410 | Both |
| [Glyoxylate and dicarboxylate metabolism](DGE---Gaojian/纹枯病DGE/upload/pathway/2-ckvs4-d.htm#gene36) | 12 | 64 | 0.0558289 | 1.81E-01 | ko00630 | Both |
| [Isoquinoline alkaloid biosynthesis](DGE---Gaojian/纹枯病DGE/upload/pathway/2-ckvs4-d.htm#gene37) | 8 | 38 | 0.0611847 | 1.87E-01 | ko00950 | Both |
| [Butanoate metabolism](DGE---Gaojian/纹枯病DGE/upload/pathway/2-ckvs4-d.htm#gene38) | 13 | 72 | 0.0618681 | 1.87E-01 | ko00650 | Both |
| [Limonene and pinene degradation](DGE---Gaojian/纹枯病DGE/upload/pathway/2-ckvs4-d.htm#gene39) | 29 | 190 | 0.062343 | 1.87E-01 | ko00903 | Both |
| [Polyketide sugar unit biosynthesis](DGE---Gaojian/纹枯病DGE/upload/pathway/2-ckvs4-d.htm#gene40) | 4 | 14 | 0.0662175 | 1.90E-01 | ko00523 | Both |
| [Fatty acid elongation in mitochondria](DGE---Gaojian/纹枯病DGE/upload/pathway/2-ckvs4-d.htm#gene41) | 2 | 4 | 0.0666244 | 1.90E-01 | ko00062 | Down-regulated |
| [Glycerolipid metabolism](DGE---Gaojian/纹枯病DGE/upload/pathway/2-ckvs4-d.htm#gene42) | 16 | 96 | 0.0763035 | 2.13E-01 | ko00561 | Both |
| [Lysine biosynthesis](DGE---Gaojian/纹枯病DGE/upload/pathway/2-ckvs4-d.htm#gene43) | 6 | 27 | 0.079321 | 2.16E-01 | ko00300 | Both |
| [alpha-Linolenic acid metabolism](DGE---Gaojian/纹枯病DGE/upload/pathway/2-ckvs4-d.htm#gene44) | 16 | 99 | 0.0947417 | 2.52E-01 | ko00592 | Both |
| [Ascorbate and aldarate metabolism](DGE---Gaojian/纹枯病DGE/upload/pathway/2-ckvs4-d.htm#gene45) | 14 | 85 | 0.1001448 | 2.56E-01 | ko00053 | Both |
| [Purine metabolism](DGE---Gaojian/纹枯病DGE/upload/pathway/2-ckvs4-d.htm#gene46) | 35 | 247 | 0.1026934 | 2.56E-01 | ko00230 | Both |
| [Protein export](DGE---Gaojian/纹枯病DGE/upload/pathway/2-ckvs4-d.htm#gene47) | 13 | 78 | 0.1027118 | 2.56E-01 | ko03060 | Both |
| [Methane metabolism](DGE---Gaojian/纹枯病DGE/upload/pathway/2-ckvs4-d.htm#gene48) | 25 | 172 | 0.1208482 | 2.90E-01 | ko00680 | Both |
| [Natural killer cell mediated cytotoxicity](DGE---Gaojian/纹枯病DGE/upload/pathway/2-ckvs4-d.htm#gene49) | 7 | 37 | 0.121641 | 2.90E-01 | ko04650 | Both |
| [Linoleic acid metabolism](DGE---Gaojian/纹枯病DGE/upload/pathway/2-ckvs4-d.htm#gene50) | 10 | 63 | 0.1759916 | 4.12E-01 | ko00591 | Both |
| [Cysteine and methionine metabolism](DGE---Gaojian/纹枯病DGE/upload/pathway/2-ckvs4-d.htm#gene51) | 28 | 206 | 0.186061 | 4.27E-01 | ko00270 | Both |
| [Propanoate metabolism](DGE---Gaojian/纹枯病DGE/upload/pathway/2-ckvs4-d.htm#gene52) | 9 | 58 | 0.2109969 | 4.68E-01 | ko00640 | Both |
| [Riboflavin metabolism](DGE---Gaojian/纹枯病DGE/upload/pathway/2-ckvs4-d.htm#gene53) | 4 | 21 | 0.2117939 | 4.68E-01 | ko00740 | Both |
| [Taurine and hypotaurine metabolism](DGE---Gaojian/纹枯病DGE/upload/pathway/2-ckvs4-d.htm#gene54) | 2 | 8 | 0.2294378 | 4.97E-01 | ko00430 | Both |
| [Alanine, aspartate and glutamate metabolism](DGE---Gaojian/纹枯病DGE/upload/pathway/2-ckvs4-d.htm#gene55) | 14 | 103 | 0.2830074 | 6.00E-01 | ko00250 | Both |
| [Selenoamino acid metabolism](DGE---Gaojian/纹枯病DGE/upload/pathway/2-ckvs4-d.htm#gene56) | 8 | 55 | 0.2871542 | 6.00E-01 | ko00450 | Both |
| [Thiamine metabolism](DGE---Gaojian/纹枯病DGE/upload/pathway/2-ckvs4-d.htm#gene57) | 3 | 17 | 0.3052148 | 6.26E-01 | ko00730 | Both |
| [Valine, leucine and isoleucine degradation](DGE---Gaojian/纹枯病DGE/upload/pathway/2-ckvs4-d.htm#gene58) | 10 | 73 | 0.3183544 | 6.32E-01 | ko00280 | Both |
| [Lysine degradation](DGE---Gaojian/纹枯病DGE/upload/pathway/2-ckvs4-d.htm#gene59) | 8 | 57 | 0.3221437 | 6.32E-01 | ko00310 | Both |
| [Galactose metabolism](DGE---Gaojian/纹枯病DGE/upload/pathway/2-ckvs4-d.htm#gene60) | 13 | 98 | 0.3242135 | 6.32E-01 | ko00052 | Both |
| [Starch and sucrose metabolism](DGE---Gaojian/纹枯病DGE/upload/pathway/2-ckvs4-d.htm#gene61) | 51 | 419 | 0.329703 | 6.32E-01 | ko00500 | Both |
| [Caffeine metabolism](DGE---Gaojian/纹枯病DGE/upload/pathway/2-ckvs4-d.htm#gene62) | 2 | 11 | 0.3621963 | 6.83E-01 | ko00232 | Up-regulated |
| [Phosphatidylinositol signaling system](DGE---Gaojian/纹枯病DGE/upload/pathway/2-ckvs4-d.htm#gene63) | 14 | 110 | 0.373222 | 6.93E-01 | ko04070 | Both |
| [Sulfur metabolism](DGE---Gaojian/纹枯病DGE/upload/pathway/2-ckvs4-d.htm#gene64) | 6 | 44 | 0.3869231 | 7.07E-01 | ko00920 | Both |
| [Steroid biosynthesis](DGE---Gaojian/纹枯病DGE/upload/pathway/2-ckvs4-d.htm#gene65) | 9 | 70 | 0.404448 | 7.28E-01 | ko00100 | Both |
| [Diterpenoid biosynthesis](DGE---Gaojian/纹枯病DGE/upload/pathway/2-ckvs4-d.htm#gene66) | 9 | 71 | 0.4213971 | 7.47E-01 | ko00904 | Both |
| [Arginine and proline metabolism](DGE---Gaojian/纹枯病DGE/upload/pathway/2-ckvs4-d.htm#gene67) | 12 | 97 | 0.428134 | 7.48E-01 | ko00330 | Both |
| [Fatty acid metabolism](DGE---Gaojian/纹枯病DGE/upload/pathway/2-ckvs4-d.htm#gene68) | 12 | 100 | 0.471533 | 8.11E-01 | ko00071 | Both |
| [Mismatch repair](DGE---Gaojian/纹枯病DGE/upload/pathway/2-ckvs4-d.htm#gene69) | 8 | 66 | 0.4838068 | 8.20E-01 | ko03430 | Both |
| [Inositol phosphate metabolism](DGE---Gaojian/纹枯病DGE/upload/pathway/2-ckvs4-d.htm#gene70) | 11 | 95 | 0.5263038 | 8.53E-01 | ko00562 | Both |
| [Histidine metabolism](DGE---Gaojian/纹枯病DGE/upload/pathway/2-ckvs4-d.htm#gene71) | 4 | 33 | 0.5285369 | 8.53E-01 | ko00340 | Both |
| [ABC transporters](DGE---Gaojian/纹枯病DGE/upload/pathway/2-ckvs4-d.htm#gene72) | 14 | 122 | 0.532301 | 8.53E-01 | ko02010 | Both |
| [Cyanoamino acid metabolism](DGE---Gaojian/纹枯病DGE/upload/pathway/2-ckvs4-d.htm#gene73) | 14 | 122 | 0.532301 | 8.53E-01 | ko00460 | Both |
| [Tryptophan metabolism](DGE---Gaojian/纹枯病DGE/upload/pathway/2-ckvs4-d.htm#gene74) | 15 | 133 | 0.5588213 | 8.84E-01 | ko00380 | Both |
| [Ubiquinone and other terpenoid-quinone biosynthesis](DGE---Gaojian/纹枯病DGE/upload/pathway/2-ckvs4-d.htm#gene75) | 8 | 73 | 0.6023987 | 9.32E-01 | ko00130 | Both |
| [Circadian rhythm - plant](DGE---Gaojian/纹枯病DGE/upload/pathway/2-ckvs4-d.htm#gene76) | 18 | 164 | 0.6053738 | 9.32E-01 | ko04712 | Both |
| [Betalain biosynthesis](DGE---Gaojian/纹枯病DGE/upload/pathway/2-ckvs4-d.htm#gene77) | 1 | 8 | 0.6204293 | 9.43E-01 | ko00965 | Up-regulated |
| [Folate biosynthesis](DGE---Gaojian/纹枯病DGE/upload/pathway/2-ckvs4-d.htm#gene78) | 2 | 19 | 0.654814 | 9.58E-01 | ko00790 | Both |
| [Brassinosteroid biosynthesis](DGE---Gaojian/纹枯病DGE/upload/pathway/2-ckvs4-d.htm#gene79) | 2 | 19 | 0.654814 | 9.58E-01 | ko00905 | Both |
| [One carbon pool by folate](DGE---Gaojian/纹枯病DGE/upload/pathway/2-ckvs4-d.htm#gene80) | 2 | 19 | 0.654814 | 9.58E-01 | ko00670 | Both |
| [SNARE interactions in vesicular transport](DGE---Gaojian/纹枯病DGE/upload/pathway/2-ckvs4-d.htm#gene81) | 7 | 69 | 0.6850406 | 9.82E-01 | ko04130 | Both |
| [Citrate cycle (TCA cycle)](DGE---Gaojian/纹枯病DGE/upload/pathway/2-ckvs4-d.htm#gene83) | 9 | 89 | 0.6985631 | 9.85E-01 | ko00020 | Both |
| [Base excision repair](DGE---Gaojian/纹枯病DGE/upload/pathway/2-ckvs4-d.htm#gene84) | 9 | 92 | 0.7356322 | 1.00E+00 | ko03410 | Both |
| [Vitamin B6 metabolism](DGE---Gaojian/纹枯病DGE/upload/pathway/2-ckvs4-d.htm#gene85) | 1 | 11 | 0.7360855 | 1.00E+00 | ko00750 | Down-regulated |
| [RNA polymerase](DGE---Gaojian/纹枯病DGE/upload/pathway/2-ckvs4-d.htm#gene86) | 8 | 84 | 0.755902 | 1.00E+00 | ko03020 | Both |
| [Peroxisome](DGE---Gaojian/纹枯病DGE/upload/pathway/2-ckvs4-d.htm#gene87) | 19 | 192 | 0.7775171 | 1.00E+00 | ko04146 | Both |
| [Aminoacyl-tRNA biosynthesis](DGE---Gaojian/纹枯病DGE/upload/pathway/2-ckvs4-d.htm#gene89) | 8 | 87 | 0.78948 | 1.00E+00 | ko00970 | Both |
| [Synthesis and degradation of ketone bodies](DGE---Gaojian/纹枯病DGE/upload/pathway/2-ckvs4-d.htm#gene90) | 1 | 13 | 0.792879 | 1.00E+00 | ko00072 | Down-regulated |
| [Anthocyanin biosynthesis](DGE---Gaojian/纹枯病DGE/upload/pathway/2-ckvs4-d.htm#gene91) | 2 | 26 | 0.8135632 | 1.00E+00 | ko00942 | Both |
| [Non-homologous end-joining](DGE---Gaojian/纹枯病DGE/upload/pathway/2-ckvs4-d.htm#gene92) | 2 | 26 | 0.8135632 | 1.00E+00 | ko03450 | Up-regulated |
| [Glycosaminoglycan degradation](DGE---Gaojian/纹枯病DGE/upload/pathway/2-ckvs4-d.htm#gene93) | 1 | 17 | 0.8724445 | 1.00E+00 | ko00531 | Down-regulated |
| [Nicotinate and nicotinamide metabolism](DGE---Gaojian/纹枯病DGE/upload/pathway/2-ckvs4-d.htm#gene94) | 1 | 17 | 0.8724445 | 1.00E+00 | ko00760 | Up-regulated |
| [Pyrimidine metabolism](DGE---Gaojian/纹枯病DGE/upload/pathway/2-ckvs4-d.htm#gene95) | 22 | 237 | 0.8744072 | 1.00E+00 | ko00240 | Both |
| [Nucleotide excision repair](DGE---Gaojian/纹枯病DGE/upload/pathway/2-ckvs4-d.htm#gene96) | 10 | 119 | 0.8843708 | 1.00E+00 | ko03420 | Both |
| [Valine, leucine and isoleucine biosynthesis](DGE---Gaojian/纹枯病DGE/upload/pathway/2-ckvs4-d.htm#gene97) | 4 | 56 | 0.8949435 | 1.00E+00 | ko00290 | Both |
| [Indole alkaloid biosynthesis](DGE---Gaojian/纹枯病DGE/upload/pathway/2-ckvs4-d.htm#gene98) | 3 | 45 | 0.9005043 | 1.00E+00 | ko00901 | Both |
| [RNA degradation](DGE---Gaojian/纹枯病DGE/upload/pathway/2-ckvs4-d.htm#gene99) | 14 | 164 | 0.9048563 | 1.00E+00 | ko03018 | Both |
| [Oxidative phosphorylation](DGE---Gaojian/纹枯病DGE/upload/pathway/2-ckvs4-d.htm#gene100) | 25 | 275 | 0.9087992 | 1.00E+00 | ko00190 | Both |
| [Homologous recombination](DGE---Gaojian/纹枯病DGE/upload/pathway/2-ckvs4-d.htm#gene101) | 6 | 81 | 0.9119765 | 1.00E+00 | ko03440 | Both |
| [Arachidonic acid metabolism](DGE---Gaojian/纹枯病DGE/upload/pathway/2-ckvs4-d.htm#gene102) | 1 | 23 | 0.9383696 | 1.00E+00 | ko00590 | Down-regulated |
| [Other glycan degradation](DGE---Gaojian/纹枯病DGE/upload/pathway/2-ckvs4-d.htm#gene103) | 2 | 40 | 0.9517173 | 1.00E+00 | ko00511 | Both |
| [Pentose and glucuronate interconversions](DGE---Gaojian/纹枯病DGE/upload/pathway/2-ckvs4-d.htm#gene104) | 10 | 136 | 0.9554722 | 1.00E+00 | ko00040 | Both |
| [Ubiquitin mediated proteolysis](DGE---Gaojian/纹枯病DGE/upload/pathway/2-ckvs4-d.htm#gene105) | 27 | 319 | 0.9650319 | 1.00E+00 | ko04120 | Both |
| [Fatty acid biosynthesis](DGE---Gaojian/纹枯病DGE/upload/pathway/2-ckvs4-d.htm#gene106) | 3 | 60 | 0.9735712 | 1.00E+00 | ko00061 | Both |
| [Carotenoid biosynthesis](DGE---Gaojian/纹枯病DGE/upload/pathway/2-ckvs4-d.htm#gene107) | 5 | 86 | 0.974039 | 1.00E+00 | ko00906 | Down-regulated |
| [Ether lipid metabolism](DGE---Gaojian/纹枯病DGE/upload/pathway/2-ckvs4-d.htm#gene108) | 2 | 47 | 0.9763291 | 1.00E+00 | ko00565 | Up-regulated |
| [Glycerophospholipid metabolism](DGE---Gaojian/纹枯病DGE/upload/pathway/2-ckvs4-d.htm#gene109) | 9 | 136 | 0.9781353 | 1.00E+00 | ko00564 | Both |
| [Plant-pathogen interaction](DGE---Gaojian/纹枯病DGE/upload/pathway/2-ckvs4-d.htm#gene110) | 121 | 1246 | 0.9795313 | 1.00E+00 | ko04626 | Both |
| [Basal transcription factors](DGE---Gaojian/纹枯病DGE/upload/pathway/2-ckvs4-d.htm#gene111) | 4 | 80 | 0.9853405 | 1.00E+00 | ko03022 | Both |
| [N-Glycan biosynthesis](DGE---Gaojian/纹枯病DGE/upload/pathway/2-ckvs4-d.htm#gene113) | 2 | 58 | 0.9925267 | 1.00E+00 | ko00510 | Down-regulated |
| [Endocytosis](DGE---Gaojian/纹枯病DGE/upload/pathway/2-ckvs4-d.htm#gene114) | 11 | 203 | 0.9989555 | 1.00E+00 | ko04144 | Both |
| [Sphingolipid metabolism](DGE---Gaojian/纹枯病DGE/upload/pathway/2-ckvs4-d.htm#gene115) | 1 | 57 | 0.9990069 | 1.00E+00 | ko00600 | Down-regulated |
| [Proteasome](DGE---Gaojian/纹枯病DGE/upload/pathway/2-ckvs4-d.htm#gene116) | 3 | 101 | 0.9995367 | 1.00E+00 | ko03050 | Up-regulated |
| Spliceosome | 32 | 462 | 0.9995953 | 1.00E+00 | ko03040 | Both |

**Table 6.list of identity of genes with different expression and the corresponding KO numbers between 4-d and 2-ck not contained in KEGG**

| Pathway | Sample1 (1639) | Sample2 (14375) | Pvalue | Qvalue | Pathway ID |
| --- | --- | --- | --- | --- | --- |
| [Metabolic pathways](file:///C:\Users\gaojian\Desktop\DGE---Gaojian\纹枯病DGE\upload\pathway\2-ckvs4-d.htm#gene4) | 445 | 3173 | 1.47E-07 | 4.31E-06 | ko01100 |
| Biosynthesis of plant hormones | 110 | 643 | 6.88E-06 | 1.34E-04 | ko01070 |
| [Biosynthesis of phenylpropanoids](file:///C:\Users\gaojian\Desktop\DGE---Gaojian\纹枯病DGE\upload\pathway\2-ckvs4-d.htm#gene7) | 126 | 762 | 8.40E-06 | 1.40E-04 | ko01061 |
| [Biosynthesis of alkaloids derived from ornithine, lysine and nicotinic acid](file:///C:\Users\gaojian\Desktop\DGE---Gaojian\纹枯病DGE\upload\pathway\2-ckvs4-d.htm#gene10) | 58 | 307 | 6.98E-05 | 8.16E-04 | ko01064 |
| [Biosynthesis of alkaloids derived from shikimate pathway](file:///C:\Users\gaojian\Desktop\DGE---Gaojian\纹枯病DGE\upload\pathway\2-ckvs4-d.htm#gene11) | 65 | 363 | 0.0001382 | 1.41E-03 | ko01063 |
| [Biosynthesis of alkaloids derived from terpenoid and polyketide](file:///C:\Users\gaojian\Desktop\DGE---Gaojian\纹枯病DGE\upload\pathway\2-ckvs4-d.htm#gene17) | 54 | 307 | 0.000759 | 5.22E-03 | ko01066 |
| [Biosynthesis of alkaloids derived from histidine and purine](file:///C:\Users\gaojian\Desktop\DGE---Gaojian\纹枯病DGE\upload\pathway\2-ckvs4-d.htm#gene25) | 44 | 272 | 0.0104652 | 4.73E-02 | ko01065 |
| [Biosynthesis of terpenoids and steroids](file:///C:\Users\gaojian\Desktop\DGE---Gaojian\纹枯病DGE\upload\pathway\2-ckvs4-d.htm#gene27) | 61 | 407 | 0.0152513 | 6.61E-02 | ko01062 |
| [Glucosinolate biosynthesis](file:///C:\Users\gaojian\Desktop\DGE---Gaojian\纹枯病DGE\upload\pathway\2-ckvs4-d.htm#gene82) | 5 | 50 | 0.6885376 | 9.82E-01 | ko00966 |
| [N-Glycan biosynthesis](file:///C:\Users\gaojian\Desktop\DGE---Gaojian\纹枯病DGE\upload\pathway\2-ckvs4-d.htm#gene113) | 2 | 58 | 0.9925267 | 1.00E+00 | ko00510 |
| [Zeatin biosynthesis](file:///C:\Users\gaojian\Desktop\DGE---Gaojian\纹枯病DGE\upload\pathway\2-ckvs4-d.htm#gene112) | 9 | 150 | 0.991677 | 1.00E+00 | ko00908 |
| [Biosynthesis of unsaturated fatty acids](file:///C:\Users\gaojian\Desktop\DGE---Gaojian\纹枯病DGE\upload\pathway\2-ckvs4-d.htm#gene88) | 8 | 87 | 0.78948 | 1.00E+00 | ko01040 |

**Table S1. List of DEGs changed for 4 fold and more in 4-d library (Continued)**

| Gene | Annotation | | | | Stress related function | | Accession | | Fold(log2 Ratio(4-d/2-ck) |
| --- | --- | --- | --- | --- | --- | --- | --- | --- | --- |
| **Defense** | | | | | | | | | |
| GRMZM2G023655 | Thaumatin, pathogenesis-related | | | | pathogen defence drought and heat combination | |  | | 4.584962501 |
| GRMZM2G393507 | Thaumatin, pathogenesis-related | | | | pathogen defence drought and heat combination | | NP_001169696 | | 6.894817763 |
| GRMZM2G136372 | Thaumatin, pathogenesis-related | | | | pathogen defence drought and heat combination | | NP_001151337 | | 9.273795599 |
| GRMZM2G039969 | Harpin-induced 1 | | | | pathogen defence senescence | | NP_001169362 | | 6.569855608 |
| GRMZM2G173163 | Harpin-induced 1 | | | | pathogen defence senescence | |  | | 4.584962501 |
| GRMZM2G069511 | Harpin-induced 1 | | | | pathogen defence senescence | | NP_001147210 | | 5.584962501 |
| GRMZM2G132777 | Glutamate 5-kinase | | | | senescence | | NP_001151983 | | 6.894817763 |
| GRMZM2G105335 | Glutamine-Leucine-Glutamine, QLQ | | | | senescence | | NP_001106022 | | 5.584962501 |
| GRMZM2G032049 | Glutamyl/glutaminyl-tRNA synthetase, class Ic | | | | senescence | | NP_001152305 | | 6.14974712 |
| GRMZM2G392125 | Beta-glucanase | | | | activation of phytoanticipins | | NP_001149692 | | 4.584962501 |
| GRMZM2G091303 | Beta-glucanase | | | | activation of phytoanticipins | |  | | 5.584962501 |
| GRMZM2G413006 | Beta-glucanase | | | | activation of phytoanticipins | | NP_001149503 | | 4.584962501 |
| GRMZM2G163574 | Flagellar hook-length control protein | | | | pathogen defence | |  | | 4.584962501 |
| GRMZM2G037146 | Flagellar hook-length control protein | | | | pathogen defence | | NP_001168326 | | 6.569855608 |
| GRMZM2G026346 | Gram-negative bacterial tonB protein | | | | pathogen defence | | NP_001146465 | | 8.744833837 |
| GRMZM2G080501 | Haem oxygenase-like, multi-helical | | | | pathogen defence | | NP_001131451 | | 6.14974712 |
| GRMZM2G047968 | Haem peroxidase, plant/fungal/bacterial | | | | pathogen defence | | NM_001176110 | | 6.569855608 |
| GRMZM2G362785 | Haem peroxidase, plant/fungal/bacterial | | | | pathogen defence | |  | | 5.584962501 |
| GRMZM2G025680 | Natural resistance-associated macrophage protein | | | | pathogen defence | |  | | 5.584962501 |
| GRMZM2G415390 | Plant disease resistance response protein | | | | pathogen defence | |  | | 4.584962501 |
| GRMZM2G420772 | Plant disease resistance response protein | | | | pathogen defence | |  | | 5.584962501 |
| GRMZM2G151425 | Universal stress protein A | | | | pathogen defence drought and heat combination | | NP_001132238 | | 11.033423 |
| GRMZM2G136662 | Actin/actin-like | | | | pathogen defence | | NM_001154181 | | 4.584962501 |
| GRMZM2G367564 | Actin/actin-like | | | | pathogen defence | |  | | 4.584962501 |
| GRMZM2G473891 | Actin/actin-like | | | | pathogen defence | |  | | 4.584962501 |
| GRMZM2G111201 | Actin-binding FH2 and DRF autoregulatory | | | | pathogen defence | |  | | 5.584962501 |
| GRMZM2G114619 | Actin-binding FH2 and DRF autoregulatory | | | | pathogen defence | | NP_001148197 | | 4.584962501 |
| GRMZM2G417410 | Actin-binding FH2 and DRF autoregulatory | | | | pathogen defence | |  | | 5.584962501 |
| GRMZM2G422190 | Actin-binding FH2 | | | | pathogen defence | |  | | 4.584962501 |
| GRMZM2G088778 | Ankyrin | | | | pathogen defence | |  | | 4.584962501 |
| GRMZM2G419563 | Ankyrin | | | | pathogen defence | |  | | 4.584962501 |
| GRMZM2G176605 | Ankyrin | | | | pathogen defence | | B6SWZ3 | | 6.894817763 |
| GRMZM2G121418 | Major facilitator superfamily, general substrate transporter | | | | pathogen defence | |  | | 4.584962501 |
| GRMZM2G012242 | Major facilitator superfamily, general substrate transporter | | | | pathogen defence | |  | | 10.45635442 |
| GRMZM2G150468 | Major facilitator superfamily, general substrate transporter | | | | pathogen defence | |  | | 9.098032083 |
| GRMZM2G166976 | Major facilitator superfamily, general substrate transporter | | | | pathogen defence | | C0P927 | | 5.584962501 |
| GRMZM2G009779 | Major facilitator superfamily, general substrate transporter | | | | pathogen defence | |  | | 6.569855608 |
| GRMZM2G063524 | Major facilitator superfamily, general substrate transporter | | | | pathogen defence | |  | | 4.584962501 |
| GRMZM2G458494 | Major facilitator superfamily, general substrate transporter | | | | pathogen defence | |  | | 4.584962501 |
| GRMZM2G033665 | Mlo-related protein | | | | pathogen defence | |  | | 5.584962501 |
| GRMZM2G175525 | Pathogenesis-related transcriptional factor and ERF, DNA-binding | | | | pathogen defence | |  | | 4.584962501 |
| GRMZM2G087059 | Pathogenesis-related transcriptional factor and ERF, DNA-binding | | | | pathogen defence | |  | | 4.584962501 |
| GRMZM2G044077 | Pathogenesis-related transcriptional factor and ERF, DNA-binding | | | | pathogen defence | |  | | 6.14974712 |
| GRMZM2G073258 | Pathogenesis-related transcriptional factor and ERF, DNA-binding | | | | pathogen defence | |  | | 4.584962501 |
| GRMZM2G123119 | Pathogenesis-related transcriptional factor and ERF, DNA-binding | | | | pathogen defence | | NP_001130815 | | 9.896332404 |
| GRMZM2G381441 | Pathogenesis-related transcriptional factor and ERF, DNA-binding | | | | pathogen defence | | NP_001170395 | | 9.381542951 |
| GRMZM2G152141 | Pectinesterase inhibitor | | | | pathogen defence | | NP_001145909 | | 4.584962501 |
| GRMZM2G439908 | Pectinesterase inhibitor | | | | pathogen defence | |  | | 6.569855608 |
| GRMZM2G082118 | Penicillin-binding protein-associated | | | | pathogen defence | | NP_001148186 | | 6.14974712 |
| GRMZM2G053384 | Pentatricopeptide repeat | | | | pathogen defence | |  | | 4.584962501 |
| GRMZM2G064501 | Pentatricopeptide repeat | | | | pathogen defence | |  | | 5.584962501 |
| GRMZM2G080898 | Pentatricopeptide repeat | | | | pathogen defence | |  | | 4.584962501 |
| GRMZM2G084176 | Pentatricopeptide repeat | | | | pathogen defence | |  | | 7.383704292 |
| GRMZM2G087495 | Pentatricopeptide repeat | | | | pathogen defence | | NP_001130303 | | 5.584962501 |
| GRMZM2G092198 | Pentatricopeptide repeat | | | | pathogen defence | |  | | 6.14974712 |
| GRMZM2G106384 | Pentatricopeptide repeat | | | | pathogen defence | | NP_001167873 | | 4.584962501 |
| GRMZM2G127015 | Pentatricopeptide repeat | | | | pathogen defence | | NP_001151863 | | 6.569855608 |
| GRMZM2G132956 | Pentatricopeptide repeat | | | | pathogen defence | |  | | 6.894817763 |
| GRMZM2G144071 | Pentatricopeptide repeat | | | | pathogen defence | | NP_001145890 | | 4.584962501 |
| GRMZM2G144142 | Pentatricopeptide repeat | | | | pathogen defence | |  | | 6.894817763 |
| GRMZM2G151975 | Pentatricopeptide repeat | | | | pathogen defence | | NP_001147934 | | 7.159871337 |
| GRMZM2G163359 | Pentatricopeptide repeat | | | | pathogen defence | |  | | 6.14974712 |
| GRMZM2G163396 | Pentatricopeptide repeat | | | | pathogen defence | |  | | 5.584962501 |
| GRMZM2G177715 | Pentatricopeptide repeat | | | | pathogen defence | |  | | 4.584962501 |
| GRMZM2G306482 | Pentatricopeptide repeat | | | | pathogen defence | |  | | 4.584962501 |
| GRMZM2G349193 | Pentatricopeptide repeat | | | | pathogen defence | | NM_001152640 | | 6.14974712 |
| GRMZM2G350410 | Pentatricopeptide repeat | | | | pathogen defence | |  | | 4.584962501 |
| GRMZM2G358381 | Pentatricopeptide repeat | | | | pathogen defence | |  | | 6.569855608 |
| GRMZM2G367234 | Pentatricopeptide repeat | | | | pathogen defence | |  | | 4.584962501 |
| GRMZM2G369792 | Pentatricopeptide repeat | | | | pathogen defence | |  | | 4.584962501 |
| GRMZM2G372632 | Pentatricopeptide repeat | | | | pathogen defence | |  | | 5.584962501 |
| GRMZM2G389645 | Pentatricopeptide repeat | | | | pathogen defence | |  | | 5.584962501 |
| GRMZM2G406746 | Pentatricopeptide repeat | | | | pathogen defence | |  | | 4.584962501 |
| GRMZM2G418749 | Pentatricopeptide repeat | | | | pathogen defence | |  | | 4.584962501 |
| GRMZM2G459532 | Pentatricopeptide repeat | | | | pathogen defence | | B6SVV0 | | 5.584962501 |
| GRMZM2G474451 | Pentatricopeptide repeat | | | | pathogen defence | |  | | 4.584962501 |
| GRMZM2G000674 | Pentatricopeptide repeat | | | | pathogen defence | |  | | 4.584962501 |
| GRMZM2G014977 | Pentatricopeptide repeat | | | | pathogen defence | | NP_001141927 | | 4.584962501 |
| GRMZM2G029859 | Pentatricopeptide repeat | | | | pathogen defence | |  | | 6.14974712 |
| GRMZM2G312201 | Pentatricopeptide repeat | | | | pathogen defence | | NM_001176556 | | 10.78381676 |
| GRMZM2G081790 | Pistil-specific extensin-like protein | | | | pathogen defence | |  | | 5.584962501 |
| GRMZM2G019807 | Pistil-specific extensin-like protein | | | | pathogen defence | | NP_001148859 | | 4.584962501 |
| GRMZM2G383790 | Pistil-specific extensin-like protein | | | | pathogen defence | |  | | 5.584962501 |
| GRMZM2G038643 | Pistil-specific extensin-like protein | | | | pathogen defence | |  | | 4.584962501 |
| **Transport** | | | | | | | | | |
| GRMZM2G027016 | Multi antimicrobial extrusion protein MatE | | | fungal resistance | | NP_001146663 | | | 4.584962501 |
| GRMZM2G078129 | Multi antimicrobial extrusion protein MatE | | | fungal resistance | |  | | | 4.584962501 |
| GRMZM2G032182 | Mitochondrial carrier protein | | | aluminum tolerance | | NP_001151324 | | | 7.159871337 |
| GRMZM2G420988 | Mitochondrial carrier protein | | | aluminum tolerance | | NP_001142273 | | | 11.17367714 |
| GRMZM2G035276 | ABC transporter, conserved site | | | Senescence drought and heat combination | |  | | | 5.584962501 |
| GRMZM2G388539 | ABC transporter, transmembrane region, type 1 | | | Senescence drought and heat combination | |  | | | 4.584962501 |
| GRMZM2G143153 | ABC transporter-like | | | Senescence drought and heat combination | |  | | | 7.577428828 |
| GRMZM2G310465 | Sugar transporter, conserved site | | | Senescence drought and heat combination | |  | | | 4.584962501 |
| GRMZM2G051894 | Sugar transporter, conserved site | | | Senescence drought and heat combination | | NP_001145721 | | | 7.383704292 |
| GRMZM2G055834 | Sugar transporter, conserved site | | | Senescence drought and heat combination | | NP_001130610 | | | 4.584962501 |
| GRMZM2G169788 | ATPase 1, copper-transporting | | | Senescence drought and heat combination | |  | | | 5.584962501 |
| GRMZM2G087974 | ATPase, AAA+ type, core | | | Senescence drought and heat combination | |  | | | 4.584962501 |
| GRMZM2G458095 | ATPase, AAA+ type, core | | | Senescence drought and heat combination | | NP_001141756 | | | 5.584962501 |
| GRMZM2G014089 | ATPase, AAA+ type, core | | | Senescence drought and heat combination | |  | | | 6.569855608 |
| GRMZM2G375807 | ATPase, AAA+ type, core | | | Senescence drought and heat combination | |  | | | 5.584962501 |
| GRMZM2G158131 | ATPase, F1 complex, OSCP/delta subunit | | | Senescence drought and heat combination | | NP_001144174 | | | 4.584962501 |
| GRMZM2G148374 | ATPase, P-type, H+ transporting proton pump | | | Senescence drought and heat combination | | NP_001169998 | | | 4.584962501 |
| GRMZM2G411940 | ATPase, P-type, K/Mg/Cd/Cu/Zn/Na/Ca/Na/H-transporter | | | Senescence drought and heat combination | |  | | | 6.14974712 |
| GRMZM2G044116 | ATPase, P-type, K/Mg/Cd/Cu/Zn/Na/Ca/Na/H-transporter | | | Senescence drought and heat combination | |  | | | 6.14974712 |
| GRMZM2G428096 | ATPase, P-type, K/Mg/Cd/Cu/Zn/Na/Ca/Na/H-transporter | | | Senescence drought and heat combination | |  | | | 4.584962501 |
| GRMZM2G395120 | ATPase, V0 complex, proteolipid subunit C | | | Senescence drought and heat combination | |  | | | 4.584962501 |
| **Transcription** | | | | | | | | | |
| GRMZM2G108418 | Leucine-rich repeat, cysteine-containing subtype | | senescence | | | | |  | 6.894817763 |
| GRMZM2G481291 | Leucine-rich repeat, cysteine-containing subtype | | senescence | | | | | NP_001147496 | 4.584962501 |
| GRMZM2G015933 | Leucine-rich repeat, N-terminal | | senescence | | | | | NP_001147358 | 7.577428828 |
| GRMZM2G100121 | Leucine-rich repeat, ribonuclease inhibitor subtype | | senescence | | | | |  | 4.584962501 |
| GRMZM2G160619 | Leucine-rich repeat, typical subtype | | senescence | | | | | NP_001170505 | 4.584962501 |
| GRMZM2G386952 | Leucine-rich repeat, typical subtype | | senescence | | | | |  | 6.14974712 |
| GRMZM2G131749 | Leucine-rich repeat, typical subtype | | senescence | | | | |  | 7.894817763 |
| GRMZM2G174128 | Leucine-rich repeat, typical subtype | | senescence | | | | |  | 7.383704292 |
| GRMZM2G333980 | Leucine-rich repeat, typical subtype | | senescence | | | | | NP_001146269 | 4.584962501 |
| GRMZM2G469313 | Leucine-rich repeat, typical subtype | | senescence | | | | |  | 5.584962501 |
| GRMZM2G440849 | Leucine-rich repeat | | senescence | | | | |  | 4.584962501 |
| GRMZM2G147966 | Calcium-binding EF-hand | | defence related senescencedrought and heat combination | | | | |  | 4.584962501 |
| GRMZM2G069922 | Calcium-binding EF-hand | | defence related senescencedrought and heat combination | | | | | NP_001141550 | 4.584962501 |
| GRMZM2G431509 | Calcium-binding EF-hand | | defence related senescencedrought and heat combination | | | | |  | 4.584962501 |
| GRMZM2G097878 | Tyrosine protein kinase | | defence related senescencedrought and heat combination | | | | | NP_001146192 | 4.584962501 |
| GRMZM2G104760 | Tyrosine protein kinase | | defence related senescencedrought and heat combination | | | | | NP_001140369 | 6.569855608 |
| GRMZM2G139223 | Tyrosine protein kinase | | defence related senescencedrought and heat combination | | | | | NP_001136610 | 4.584962501 |
| GRMZM2G152901 | Tyrosine protein kinase | | defence related senescencedrought and heat combination | | | | |  | 5.584962501 |
| GRMZM2G301512 | Tyrosine protein kinase | | defence related senescencedrought and heat combination | | | | |  | 5.584962501 |
| GRMZM2G472966 | Tyrosine protein kinase | | defence related senescencedrought and heat combination | | | | |  | 5.584962501 |
| GRMZM2G089461 | Tyrosine protein kinase | | defence related senescencedrought and heat combination | | | | | B8A1J0 | 4.584962501 |
| GRMZM2G089819 | Tyrosine protein kinase | | defence related senescencedrought and heat combination | | | | | NP_001147803 | 4.584962501 |
| GRMZM2G103070 | Tyrosine protein kinase | | defence related senescencedrought and heat combination | | | | |  | 7.159871337 |
| GRMZM2G456669 | Tyrosine protein kinase | | defence related senescencedrought and heat combination | | | | |  | 6.569855608 |
| GRMZM2G003602 | Tyrosine protein kinase | | defence related senescencedrought and heat combination | | | | |  | 7.383704292 |
| GRMZM2G075247 | Tyrosine protein kinase | | defence related senescencedrought and heat combination | | | | |  | 6.569855608 |
| GRMZM2G084791 | Tyrosine protein kinase | | defence related senescencedrought and heat combination | | | | |  | 7.383704292 |
| GRMZM2G107645 | Tyrosine protein kinase | | defence related senescencedrought and heat combination | | | | | NM_001157125 | 4.584962501 |
| GRMZM2G146794 | Tyrosine protein kinase | | defence related senescencedrought and heat combination | | | | | NP_001152329 | 7.159871337 |
| GRMZM2G383881 | Tyrosine protein kinase | | defence related senescencedrought and heat combination | | | | |  | 6.569855608 |
| GRMZM2G090725 | Tyrosine protein kinase | | defence related senescencedrought and heat combination | | | | |  | 4.584962501 |
| GRMZM2G092604 | Tyrosine protein kinase | | defence related senescencedrought and heat combination | | | | |  | 6.894817763 |
| GRMZM2G130213 | Tyrosine protein kinase | | defence related senescencedrought and heat combination | | | | |  | 6.14974712 |
| GRMZM2G142390 | Tyrosine protein kinase | | defence related senescencedrought and heat combination | | | | |  | 5.584962501 |
| GRMZM2G303965 | Tyrosine protein kinase | | defence related senescencedrought and heat combination | | | | |  | 6.569855608 |
| GRMZM2G316907 | Tyrosine protein kinase | | defence related senescencedrought and heat combination | | | | |  | 4.584962501 |
| GRMZM2G332280 | Tyrosine protein kinase | | defence related senescencedrought and heat combination | | | | |  | 4.584962501 |
| GRMZM2G340654 | Tyrosine protein kinase | | defence related senescencedrought and heat combination | | | | |  | 6.569855608 |
| GRMZM2G357873 | Tyrosine protein kinase | | defence related senescencedrought and heat combination | | | | | NP_001130373 | 4.584962501 |
| GRMZM2G465987 | Tyrosine protein kinase | | defence related senescencedrought and heat combination | | | | |  | 4.584962501 |
| GRMZM2G481531 | Tyrosine protein kinase | | defence related senescencedrought and heat combination | | | | |  | 4.584962501 |
| GRMZM2G540772 | Tyrosine protein kinase | | defence related senescencedrought and heat combination | | | | |  | 7.383704292 |
| GRMZM2G007477 | Tyrosine protein kinase | | defence related senescencedrought and heat combination | | | | |  | 4.584962501 |
| GRMZM2G009166 | Tyrosine protein kinase | | defence related senescencedrought and heat combination | | | | |  | 4.584962501 |
| GRMZM2G022514 | Tyrosine protein kinase | | defence related senescencedrought and heat combination | | | | |  | 4.584962501 |
| GRMZM2G039934 | Tyrosine protein kinase | | defence related senescencedrought and heat combination | | | | | NP_001169541 | 4.584962501 |
| GRMZM2G048801 | Tyrosine protein kinase | | defence related senescencedrought and heat combination | | | | |  | 7.159871337 |
| GRMZM2G098828 | Tyrosine protein kinase | | defence related senescencedrought and heat combination | | | | |  | 5.584962501 |
| GRMZM2G112309 | Tyrosine protein kinase | | defence related senescencedrought and heat combination | | | | |  | 7.383704292 |
| GRMZM2G145753 | Tyrosine protein kinase | | defence related senescencedrought and heat combination | | | | | NP_001142419 | 4.584962501 |
| GRMZM2G146866 | Tyrosine protein kinase | | defence related senescencedrought and heat combination | | | | |  | 7.159871337 |
| GRMZM2G168985 | Tyrosine protein kinase | | defence related senescencedrought and heat combination | | | | |  | 4.584962501 |
| GRMZM2G303909 | Tyrosine protein kinase | | defence related senescencedrought and heat combination | | | | |  | 6.569855608 |
| GRMZM2G308365 | Tyrosine protein kinase | | defence related senescencedrought and heat combination | | | | |  | 6.14974712 |
| GRMZM2G337766 | Tyrosine protein kinase | | defence related senescencedrought and heat combination | | | | |  | 4.584962501 |
| GRMZM2G341302 | Tyrosine protein kinase | | defence related senescencedrought and heat combination | | | | |  | 4.584962501 |
| GRMZM2G341379 | Tyrosine protein kinase | | defence related senescencedrought and heat combination | | | | |  | 5.584962501 |
| GRMZM2G353726 | Tyrosine protein kinase | | defence related senescencedrought and heat combination | | | | |  | 6.14974712 |
| GRMZM2G422340 | Tyrosine protein kinase | | defence related senescencedrought and heat combination | | | | |  | 5.584962501 |
| GRMZM2G436448 | Tyrosine protein kinase | | defence related senescencedrought and heat combination | | | | |  | 8.033423002 |
| GRMZM2G451007 | Tyrosine protein kinase | | defence related senescencedrought and heat combination | | | | |  | 7.159871337 |
| GRMZM2G072292 | Tyrosine protein kinase | | defence related senescencedrought and heat combination | | | | | NP_001132090 | 4.584962501 |
| GRMZM2G462623 | Transcription factor E2F/dimerisation partner (TDP) | | defence, stresses | | | | |  | 4.584962501 |
| GRMZM2G398135 | Transcription factor TFIIB related | | defence, stresses | | | | |  | 4.584962501 |
| GRMZM2G099577 | Transcription factor, MADS-box | | defence, stresses | | | | | NP_001148502 | 4.584962501 |
| GRMZM2G180168 | Transcriptional factor B3 | | defence, stresses | | | | |  | 4.584962501 |
| GRMZM2G181254 | Transcriptional factor B3 | | defence, stresses | | | | | NP_001130477 | 4.584962501 |
| GRMZM2G320754 | Transcriptional factor B3 | | defence, stresses | | | | |  | 6.14974712 |
| GRMZM2G361376 | Transcriptional factor B3 | | defence, stresses | | | | |  | 5.584962501 |
| GRMZM2G026171 | Zinc finger, C2H2-like | | defence, stresses | | | | |  | 7.159871337 |
| GRMZM2G069176 | Zinc finger, C2H2-like | | defence, stresses | | | | |  | 8.159871337 |
| GRMZM2G159402 | Zinc finger, C2H2-type | | defence, stresses | | | | | Q6QP57 | 7.159871337 |
| GRMZM2G113860 | Zinc finger, C2H2-type | | defence, stresses | | | | |  | 10.033423 |
| GRMZM2G133552 | Zinc finger, C2H2-type | | defence, stresses | | | | |  | 6.569855608 |
| GRMZM2G390009 | Zinc finger, C6HC-type | | defence, stresses | | | | |  | 4.584962501 |
| GRMZM2G093404 | Zinc finger, CCCH-type | | defence, stresses | | | | | NP_001152728 | 9.743151394 |
| GRMZM2G021834 | Zinc finger, CCCH-type | | defence, stresses | | | | | NP_001168741 | 7.894817763 |
| GRMZM2G406715 | Zinc finger, CCHC-type | | defence, stresses | | | | |  | 6.569855608 |
| GRMZM2G005834 | Zinc finger, DHHC-type | | defence, stresses | | | | |  | 7.741466986 |
| GRMZM2G347488 | Zinc finger, FYVE/PHD-type | | defence, stresses | | | | | NP_001143037 | 6.569855608 |
| GRMZM2G305124 | Zinc finger, FYVE/PHD-type | | defence, stresses | | | | |  | 6.894817763 |
| GRMZM2G158194 | Zinc finger, PHD-type | | defence, stresses | | | | |  | 4.584962501 |
| GRMZM2G044550 | Zinc finger, RanBP2-type | | defence, stresses | | | | | NP_001130134 | 6.14974712 |
| GRMZM2G000353 | Zinc finger, RING-CH-type | | defence, stresses | | | | |  | 4.584962501 |
| GRMZM2G004480 | Zinc finger, RING-CH-type | | defence, stresses | | | | |  | 4.584962501 |
| GRMZM2G016362 | Zinc finger, RING-CH-type | | defence, stresses | | | | |  | 6.14974712 |
| GRMZM2G026562 | Zinc finger, RING-CH-type | | defence, stresses | | | | |  | 6.14974712 |
| GRMZM2G044537 | Zinc finger, RING-CH-type | | defence, stresses | | | | | NP_001147218 | 4.584962501 |
| GRMZM2G075782 | Zinc finger, RING-CH-type | | defence, stresses | | | | | C4J874 | 4.584962501 |
| GRMZM2G099850 | Zinc finger, RING-CH-type | | defence, stresses | | | | | NP_001141278 | 4.584962501 |
| GRMZM2G166278 | Zinc finger, RING-CH-type | | defence, stresses | | | | |  | 4.584962501 |
| GRMZM2G460958 | Zinc finger, RING-CH-type | | defence, stresses | | | | |  | 6.569855608 |
| GRMZM2G023755 | Zinc finger, RING-CH-type | | defence, stresses | | | | | NP_001147246 | 4.584962501 |
| GRMZM2G061704 | Zinc finger, RING-type, conserved site | | defence, stresses | | | | |  | 7.159871337 |
| GRMZM2G018241 | Zinc finger, RING-type | | defence, stresses | | | | | NP_001104959 | 4.584962501 |
| GRMZM2G066169 | Zinc finger, RING-type | | defence, stresses | | | | | NP_001130455 | 4.584962501 |
| GRMZM2G144101 | Zinc finger, Tim10/DDP-type | | defence, stresses | | | | | NP_001141167 | 6.894817763 |
| GRMZM2G139029 | Basic helix-loop-helix dimerisation region bHLH | | senescence | | | | | NP_001142297 | 7.159871337 |
| GRMZM2G175480 | Basic helix-loop-helix dimerisation region bHLH | | senescence | | | | |  | 6.14974712 |
| GRMZM2G178182 | Basic helix-loop-helix dimerisation region bHLH | | senescence | | | | | NP_001132785 | 4.584962501 |
| GRMZM2G378653 | Basic helix-loop-helix dimerisation region bHLH | | senescence | | | | | NP_001141213 | 6.569855608 |
| GRMZM2G030877 | Basic-leucine zipper (bZIP) transcription factor | | senescence | | | | | NP_001169220 | 4.584962501 |
| GRMZM2G036703 | DNA-binding WRKY | | senescence,stresses | | | | |  | 4.584962501 |
| GRMZM2G099593 | DNA-binding WRKY | | senescence,stresses | | | | |  | 4.584962501 |
| GRMZM2G101405 | DNA-binding WRKY | | senescence,stresses | | | | |  | 6.14974712 |
| GRMZM2G403181 | DNA-binding WRKY | | senescence,stresses | | | | |  | 5.584962501 |
| GRMZM2G432583 | DNA-binding WRKY | | senescence,stresses | | | | | NP_001143650 | 4.584962501 |
| GRMZM2G462623 | Transcription factor E2F/dimerisation partner (TDP)IPR014889 | | senescence,stresses | | | | |  | 4.584962501 |
| GRMZM2G398135 | Transcription factor TFIIB relatedIPR011028 | | senescence,stresses | | | | |  | 4.584962501 |
| ***Signal transduction*** | | | | | | | | | |
| GRMZM2G048131 | AUX/IAA protein | defence related senescencedrought and heat combination | | | | NP_001147177 | | | 4.584962501 |
| GRMZM2G074742 | AUX/IAA protein | defence related senescencedrought and heat combination | | | |  | | | 5.584962501 |
| GRMZM2G121309 | AUX/IAA protein | defence related senescencedrought and heat combination | | | | NM_001156253 | | | 4.584962501 |
| GRMZM2G163848 | AUX/IAA protein | defence related senescencedrought and heat combination | | | | NP_001132531 | | | 6.14974712 |
| GRMZM2G089640 | Auxin response factor | defence related senescencedrought and heat combination | | | |  | | | 4.584962501 |
| GRMZM2G039776 | Auxin responsive SAUR protein | defence related senescencedrought and heat combination | | | |  | | | 5.584962501 |
| GRMZM2G076345 | Auxin responsive SAUR protein | defence related senescencedrought and heat combination | | | | NP_001152632 | | | 5.584962501 |
| GRMZM2G332390 | Auxin responsive SAUR protein | defence related senescencedrought and heat combination | | | | NP_001147394 | | | 10.76404222 |
| GRMZM2G399644 | Auxin responsive SAUR protein | defence related senescencedrought and heat combination | | | |  | | | 4.584962501 |
| GRMZM2G422419 | Auxin responsive SAUR protein | defence related senescencedrought and heat combination | | | |  | | | 4.584962501 |
| GRMZM2G078508 | Auxin-binding protein | defence related senescencedrought and heat combination | | | | P33489 | | | 4.584962501 |
| GRMZM2G082630 | Helix-loop-helix DNA-binding | drought and heat combination | | | |  | | | 4.584962501 |
| GRMZM2G094892 | Helix-loop-helix DNA-binding | drought and heat combination | | | | NP_001169147 | | | 4.584962501 |
| GRMZM2G159937 | Helix-loop-helix DNA-binding | drought and heat combination | | | | NP_001150726 | | | 6.14974712 |
| GRMZM2G340177 | Helix-loop-helix DNA-binding | drought and heat combination | | | |  | | | 9.965784285 |
| GRMZM2G397755 | Helix-loop-helix DNA-binding | drought and heat combination | | | |  | | | 11.39392668 |
| GRMZM2G042920 | Helix-loop-helix DNA-binding | drought and heat combination | | | | NP_001140849 | | | 5.584962501 |
| GRMZM2G104204 | Helix-turn-helix motif, lambda-like repressor | drought and heat combination | | | |  | | | 6.894817763 |
| GRMZM2G047715 | Helix-turn-helix motif, lambda-like repressor | drought and heat combination | | | |  | | | 6.14974712 |
| GRMZM2G028041 | Helix-turn-helix motif, lambda-like repressor | drought and heat combination | | | | Q41853 | | | 4.584962501 |
| GRMZM2G087741 | Helix-turn-helix motif, lambda-like repressor | drought and heat combination | | | | P56669 | | | 5.584962501 |
| GRMZM2G041729 | Calcium-binding EF hand | drought and heat combination | | | | C3TRS1 | | | 5.584962501 |
| GRMZM2G419452 | Calcium-binding EF hand | drought and heat combination | | | | B6TUX1 | | | 4.584962501 |
| GRMZM2G040094 | Calcium-binding EF-HAND 1 | drought and heat combination | | | | NP_001170273 | | | 4.584962501 |
| GRMZM2G366411 | Calcium-binding EF-HAND 1 | drought and heat combination | | | |  | | | 4.584962501 |
| GRMZM2G001814 | Calcium-binding EF-HAND 1 | drought and heat combination | | | |  | | | 4.584962501 |
| GRMZM2G021998 | Calcium-binding EF-HAND 1 | drought and heat combination | | | | NP_001140968 | | | 4.584962501 |
| GRMZM2G080889 | Ubiquitin system component Cue | defence related senescencedrought and heat combination | | | | C0HGH2 | | | 4.584962501 |
| GRMZM2G377553 | Ubiquitin | defence related senescencedrought and heat combination | | | | NP_001159154 | | | 4.584962501 |
| GRMZM2G168829 | Ubiquitin | defence related senescencedrought and heat combination | | | |  | | | 4.584962501 |
| GRMZM2G030768 | Ubiquitin-associated/translation elongation factor EF1B, N-terminal, eukaryote | defence related senescencedrought and heat combination | | | | NP_001169906 | | | 7.741466986 |
| GRMZM2G102421 | Ubiquitin-conjugating enzyme, E2 | defence related senescencedrought and heat combination | | | | NP_001152661 | | | 4.584962501 |
| GRMZM2G002830 | Ubiquitin-conjugating enzyme, E2 | defence related senescencedrought and heat combination | | | | NP_001149453 | | | 4.584962501 |
| GRMZM2G085849 | Ubiquitin-conjugating enzyme, E2 | defence related senescencedrought and heat combination | | | | NP_001105287 | | | 4.584962501 |
| GRMZM2G116919 | Ubiquitin-conjugating enzyme, E2 | defence related senescencedrought and heat combination | | | | NP_001132346 | | | 6.14974712 |
| **Metabolism** | | | | | | | | | |
| GRMZM2G119766 | Phosphate-induced protein 1 conserved region | | | biotic and abotic stresses | |  | | | 4.584962501 |
| GRMZM2G039009 | SAM dependent carboxyl methyltransferase | | | biotic and abotic stresses | |  | | | 7.383704292 |
| GRMZM2G039993 | SAM dependent carboxyl methyltransferase | | | biotic and abotic stresses | | C4J4Q4 | | | 4.584962501 |
| GRMZM2G300965 | Cytochrome b245, heavy chain | | | Senescence drought and heat combination | | NP_001167766 | | | 11.3553511 |
| GRMZM2G031938 | Cytochrome c oxidase, subunit | | | Senescence drought and heat combination | | NP_001142368 | | | 4.584962501 |
| GRMZM2G350284 | Cytochrome c-type biogenesis protein CcmB | | | Senescence drought and heat combination | |  | | | 4.584962501 |
| GRMZM2G096909 | Cytochrome c-type biogenesis protein CcmF | | | Senescence drought and heat combination | | NP_001130415 | | | 5.584962501 |
| GRMZM2G027797 | Cytochrome P450, conserved site | | | Senescence drought and heat combination | | NP_001141446 | | | 5.584962501 |
| GRMZM2G159179 | Cytochrome P450, C-terminal region | | | Senescence drought and heat combination | |  | | | 6.894817763 |
| GRMZM2G424804 | Cytochrome P450, C-terminal region | | | Senescence drought and heat combination | | C4J647 | | | 9.159871337 |
| GRMZM2G013357 | Cytochrome P450, E-class, group | | | Senescence drought and heat combination | | NP_001131179 | | | 6.14974712 |
| GRMZM2G067591 | Cytochrome P450, E-class, group | | | Senescence drought and heat combination | | NM_001147894 | | | 8.159871337 |
| GRMZM2G096086 | Cytochrome P450, E-class, group | | | Senescence drought and heat combination | |  | | | 4.584962501 |
| GRMZM2G147245 | Cytochrome P450, E-class, group | | | Senescence drought and heat combination | | NP_001140681 | | | 6.569855608 |
| GRMZM2G167698 | Cytochrome P450, E-class, group | | | Senescence drought and heat combination | |  | | | 4.584962501 |
| GRMZM2G178351 | Cytochrome P450, E-class, group | | | Senescence drought and heat combination | | B6SYQ1 | | | 6.14974712 |
| GRMZM2G399530 | Cytochrome P450, E-class, group | | | Senescence drought and heat combination | |  | | | 6.894817763 |
| GRMZM2G401463 | Cytochrome P450, E-class, group | | | Senescence drought and heat combination | | NP_001169303 | | | 7.159871337 |
| GRMZM2G407650 | Cytochrome P450, E-class, group | | | Senescence drought and heat combination | |  | | | 4.584962501 |
| GRMZM2G455017 | Cytochrome P450, E-class, group | | | Senescence drought and heat combination | |  | | | 4.584962501 |
| GRMZM2G066441 | Cytochrome P450, E-class, group | | | Senescence drought and heat combination | | NP_001169224 | | | 7.383704292 |
| GRMZM2G122654 | Cytochrome P450, E-class, group | | | Senescence drought and heat combination | | NP_001142188 | | | 8.379378367 |
| GRMZM2G134597 | Cytochrome P450, E-class, group | | | Senescence drought and heat combination | |  | | | 7.159871337 |
| GRMZM2G147752 | Cytochrome P450, E-class, group | | | Senescence drought and heat combination | | C0P7A4 | | | 8.744833837 |
| GRMZM2G147774 | Cytochrome P450, E-class, group | | | Senescence drought and heat combination | |  | | | 7.383704292 |
| GRMZM2G150907 | Cytochrome P450, E-class, group | | | Senescence drought and heat combination | | NP_001140247 | | | 6.569855608 |
| GRMZM2G159353 | Cytochrome P450, E-class, group | | | Senescence drought and heat combination | | NP_001132688 | | | 10.033423 |
| GRMZM2G312069 | Cytochrome P450, E-class, group | | | Senescence drought and heat combination | | NP_001142304 | | | 4.584962501 |
| GRMZM2G079298 | Cytochrome P450 | | | Senescence drought and heat combination | |  | | | 4.584962501 |
| GRMZM2G479125 | Protein of unknown function DUF1751, integral membrane | | | Senescence drought and heat combination | |  | | | 4.584962501 |
| GRMZM2G022740 | Plant peroxidase | | | biotic and abotic stresses | |  | | | 8.159871337 |
| GRMZM2G025441 | Plant peroxidase | | | biotic and abotic stresses | | A5H452 | | | 6.894817763 |
| GRMZM2G029144 | Plant peroxidase | | | biotic and abotic stresses | |  | | | 6.894817763 |
| GRMZM2G057487 | Plant peroxidase | | | biotic and abotic stresses | |  | | | 5.584962501 |
| GRMZM2G076562 | Plant peroxidase | | | biotic and abotic stresses | | NP_001136740 | | | 8.276124405 |
| GRMZM2G117365 | Plant peroxidase | | | biotic and abotic stresses | |  | | | 4.584962501 |
| GRMZM2G136525 | Plant peroxidase | | | biotic and abotic stresses | | NP_001132283 | | | 4.584962501 |
| GRMZM2G320269 | Plant peroxidase | | | biotic and abotic stresses | | NP_001148726 | | | 5.584962501 |
| GRMZM2G410175 | Plant peroxidase | | | biotic and abotic stresses | | D7NLB3 | | | 5.584962501 |
| GRMZM2G041685 | Glutathione S-transferase, C-terminal-like | | | biotic and abotic stresses | | NM_001111522 | | | 7.383704292 |
| GRMZM2G156877 | Glutathione S-transferase, C-terminal-like | | | biotic and abotic stresses | | NP_001105720 | | | 10.09803208 |
| GRMZM2G032856 | Glutathione S-transferase, C-terminal-like | | | biotic and abotic stresses | | NP_001104988 | | | 5.584962501 |
| GRMZM2G129357 | Glutathione S-transferase, C-terminal-like | | | biotic and abotic stresses | | NP_001146786 | | | 8.033423002 |
| GRMZM2G003489 | Heat shock factor (HSF)-type, DNA-binding | | | biotic and abotic stresses | |  | | | 7.159871337 |
| GRMZM2G025685 | Heat shock factor (HSF)-type, DNA-binding | | | biotic and abotic stresses | | NM_001150893 | | | 5.584962501 |
| GRMZM2G330457 | Heat shock protein DnaJ, N-terminal | | | biotic and abotic stresses | |  | | | 4.584962501 |
| GRMZM2G434839 | Heat shock protein DnaJ | | | biotic and abotic stresses | |  | | | 4.584962501 |
| GRMZM2G135608 | Heat shock protein Hsp70 | | | biotic and abotic stresses | | C4J3B5 | | | 6.14974712 |
| GRMZM2G310431 | Heat shock protein Hsp70 | | | biotic and abotic stresses | | P11143 | | | 5.584962501 |
| GRMZM2G471196 | Heat shock protein Hsp70 | | | biotic and abotic stresses | |  | | | 5.584962501 |
| GRMZM2G306679 | HSP20-like chaperone | | | biotic and abotic stresses | | NP_001130454 | | | 4.584962501 |
| GRMZM2G331701 | HSP20-like chaperone | | | biotic and abotic stresses | | NP_001151139 | | | 4.584962501 |
| GRMZM2G479260 | HSP20-like chaperone | | | biotic and abotic stresses | | NP_001150137 | | | 6.14974712 |
| GRMZM2G089147 | HSP20-like chaperone | | | biotic and abotic stresses | | NP_001130083 | | | 4.584962501 |
| GRMZM2G007795 | UDP-glucuronosyl/UDP-glucosyltransferase | | | biotic and abotic stresses | | NP_001148283 | | | 4.584962501 |
| GRMZM2G035755 | UDP-glucuronosyl/UDP-glucosyltransferase | | | biotic and abotic stresses | | NP_001140972 | | | 6.14974712 |
| GRMZM2G036409 | UDP-glucuronosyl/UDP-glucosyltransferase | | | biotic and abotic stresses | | NM_001155095 | | | 8.965784285 |
| GRMZM2G051683 | UDP-glucuronosyl/UDP-glucosyltransferase | | | biotic and abotic stresses | | NP_001159290 | | | 6.894817763 |
| GRMZM2G067361 | UDP-glucuronosyl/UDP-glucosyltransferase | | | biotic and abotic stresses | | NP_001168299 | | | 4.584962501 |
| GRMZM2G095261 | UDP-glucuronosyl/UDP-glucosyltransferase | | | biotic and abotic stresses | |  | | | 6.569855608 |
| GRMZM2G099740 | UDP-glucuronosyl/UDP-glucosyltransferase | | | biotic and abotic stresses | | NP_001137048 | | | 4.584962501 |
| GRMZM2G130119 | UDP-glucuronosyl/UDP-glucosyltransferase | | | biotic and abotic stresses | |  | | | 8.965784285 |
| GRMZM2G301148 | UDP-glucuronosyl/UDP-glucosyltransferase | | | biotic and abotic stresses | |  | | | 4.584962501 |
| GRMZM2G304712 | UDP-glucuronosyl/UDP-glucosyltransferase | | | biotic and abotic stresses | | NP_001147693 | | | 7.894817763 |
| GRMZM2G325023 | UDP-glucuronosyl/UDP-glucosyltransferase | | | biotic and abotic stresses | | NP_001141161 | | | 6.14974712 |
| GRMZM2G395508 | UDP-glucuronosyl/UDP-glucosyltransferase | | | biotic and abotic stresses | |  | | | 4.584962501 |
| GRMZM2G426242 | UDP-glucuronosyl/UDP-glucosyltransferase | | | biotic and abotic stresses | |  | | | 7.159871337 |
| GRMZM2G458776 | UDP-glucuronosyl/UDP-glucosyltransferase | | | biotic and abotic stresses | |  | | | 8.479780264 |
| GRMZM2G463996 | UDP-glucuronosyl/UDP-glucosyltransferase | | | biotic and abotic stresses | |  | | | 4.584962501 |
| GRMZM2G475884 | UDP-glucuronosyl/UDP-glucosyltransferase | | | biotic and abotic stresses | |  | | | 5.584962501 |
| GRMZM2G354456 | UDP-glucuronosyl/UDP-glucosyltransferase | | | biotic and abotic stresses | |  | | | 5.584962501 |
| GRMZM2G372068 | UDP-glucuronosyl/UDP-glucosyltransferase | | | biotic and abotic stresses | | NP_001130518 | | | 8.379378367 |
| GRMZM2G075140 | Thiolase-like | | | biotic and abotic stresses | |  | | | 4.584962501 |
| GRMZM2G111677 | Thiolase-like | | | biotic and abotic stresse | |  | | | 5.584962501 |
| GRMZM2G033489 | Thionin | | | biotic and abotic stresse | | NP_001141901 | | | 4.584962501 |
| GRMZM2G321606 | Thioredoxin-like fold | | | biotic and abotic stresse | |  | | | 7.159871337 |
| GRMZM2G034283 | Thioredoxin-like fold | | | biotic and abotic stresse | | C4J1U6 | | | 6.14974712 |
| GRMZM2G465689 | Thioredoxin-like fold | | | biotic and abotic stresse | |  | | | 6.894817763 |
| GRMZM2G075140 | Thiolase-like | | | biotic and abotic stresse | |  | | | 4.584962501 |
| GRMZM2G111677 | Thiolase-like | | | biotic and abotic stresse | |  | | | 5.584962501 |
| GRMZM2G015908 | Pyridoxal phosphate phosphatase-relate | | | defence related | | NP_001151156 | | | 7.383704292 |
| GRMZM2G034690 | Pyridoxal phosphate-dependent transferase | | | defence related | | NP_001169374 | | | 5.584962501 |
| GRMZM2G175396 | Pyridoxal phosphate-dependent transferase, | | | defence related | | NP_001146481 | | | 6.569855608 |
| GRMZM2G147268 | Pyridoxal phosphate-dependent transferase, | | | defence related | | NP_001141974 | | | 4.584962501 |
| GRMZM2G124820 | Pyruvate/Phosphoenolpyruvate kinase, | | | defence related | | NP_001140413 | | | 5.584962501 |
| GRMZM2G015908 | Pyridoxal phosphate phosphatase-related | | | defence related | | NP_001151156 | | | 7.383704292 |
| GRMZM2G034690 | Pyridoxal phosphate-dependent transferase, | | | defence related | | NP_001169374 | | | 5.584962501 |
| GRMZM2G175396 | Pyridoxal phosphate-dependent transferase, | | | defence related | | NP_001146481 | | | 6.569855608 |
| GRMZM2G147268 | Pyridoxal phosphate-dependent transferase, | | | defence related | | NP_001141974 | | | 4.584962501 |
| GRMZM2G124820 | Pyruvate/Phosphoenolpyruvate kinase, | | | defence related | | NP_001140413 | | | 5.584962501 |
| GRMZM2G015908 | Pyridoxal phosphate phosphatase-related | | | defence related | | NP_001151156 | | | 7.383704292 |
| GRMZM2G122228 | Protein phosphatase 2C, manganese/magnesium aspartate binding site | | | defence related senescencedrought | | B4FW20 | | | 5.584962501 |
| GRMZM2G407623 | Protein phosphatase 2C-related | | | defence related senescencedrought | |  | | | 4.584962501 |
| GRMZM2G122228 | Protein phosphatase 2C, manganese/magnesium aspartate binding site | | | defence related senescencedrought | | B4FW20 | | | 5.584962501 |
| GRMZM2G407623 | Protein phosphatase 2C-related; | | | defence related senescencedrought | |  | | | 4.584962501 |
| GRMZM2G122228 | Protein phosphatase 2C, manganese/magnesium aspartate binding site; | | | defence related senescencedrought | | B4FW20 | | | 5.584962501 |
| GRMZM2G017557 | Protein kinase, ATP binding site | | | defence related | | P47917 | | | 4.584962501 |
| GRMZM2G172368 | Protein kinase, ATP binding site; | | | defence related | |  | | | 6.14974712 |
| GRMZM2G013767 | Protein kinase, ATP binding site; | | | defence related | |  | | | 5.584962501 |
| GRMZM2G080871 | Protein kinase, ATP binding site; | | | defence related | |  | | | 4.584962501 |
| GRMZM2G052386 | Protein kinase, ATP binding site | | | defence related | |  | | | 6.569855608 |
| GRMZM2G127984 | Protein kinase-like | | | defence related | | NP_001140282 | | | 5.584962501 |
| GRMZM2G324727 | Protein kinase-like | | | defence related | |  | | | 4.584962501 |
| GRMZM2G094541 | Protein kinase-like; | | | defence related | | NP_001141681 | | | 5.584962501 |
| GRMZM2G134332 | Protein kinase-like; | | | defence related | | NP_001130351 | | | 4.584962501 |
| GRMZM2G017557 | Protein kinase, ATP binding site; | | | defence related | | P47917 | | | 4.584962501 |
| GRMZM2G172368 | Protein kinase, ATP binding site; | | | defence related | |  | | | 6.14974712 |
| GRMZM2G013767 | Protein kinase, ATP binding site; | | | defence related | |  | | | 5.584962501 |
| GRMZM2G080871 | Protein kinase, ATP binding site; | | | defence related | |  | | | 4.584962501 |
| GRMZM2G052386 | Protein kinase, ATP binding site; | | | defence related | |  | | | 6.569855608 |
| GRMZM2G127984 | Protein kinase-like | | | defence related | | NP_001140282 | | | 5.584962501 |
| GRMZM2G009510 | Polyketide synthase, type III; | | | defence related | |  | | | 4.584962501 |
| GRMZM2G027130 | Polyketide synthase, type III; | | | defence related | |  | | | 4.584962501 |
| GRMZM2G108894 | Polyketide synthase, type III | | | defence related | | NP_001149508 | | | 4.584962501 |
| GRMZM2G419182 | Polynucleotidyl transferase, Ribonuclease H fold; | | | defence, stresses | |  | | | 6.894817763 |
| GRMZM2G175236 | Polynucleotidyl transferase, Ribonuclease H fold | | | defence, stresses | | NP_001151909 | | | 4.584962501 |
| GRMZM2G047019 | Polynucleotidyl transferase, Ribonuclease H fold | | | defence, stresses | | NP_001140241 | | | 6.569855608 |
| GRMZM2G130634 | Polynucleotidyl transferase, Ribonuclease H fold; | | | defence, stresses | | B6UE98 | | | 4.584962501 |
| GRMZM2G009510 | Polyketide synthase, type III | | | defence related | |  | | | 4.584962501 |
| GRMZM2G027130 | Polyketide synthase, type III | | | defence related | |  | | | 4.584962501 |
| GRMZM2G439268 | Plant lipid transfer protein/Par allergen | | | defence related | |  | | | 5.584962501 |
| GRMZM2G027167 | Plant lipid transfer protein/seed storage/trypsin-alpha amylase inhibitor | | | defence related | |  | | | 4.584962501 |
| GRMZM2G423331 | Plant methyltransferase dimerisation | | | defence related | |  | | | 4.584962501 |
| GRMZM2G081585 | Manganese and iron superoxide dismutase; | | | defence related | | NP_001132167 | | | 4.584962501 |
| GRMZM2G124455 | Manganese and iron superoxide dismutase; | | | defence related | | P41980 | | | 5.584962501 |
| GRMZM2G053394 | IQ calmodulin-binding region; | | | defence, stresses | |  | | | 4.584962501 |
| GRMZM2G382918 | IQ calmodulin-binding region; | | | defence, stresses | |  | | | 4.584962501 |
| GRMZM2G031432 | Isopenicillin N synthase; | | | defence, stresses | | NP_001150737 | | | 6.894817763 |
| GRMZM2G031724 | Isopenicillin N synthase; | | | defence, stresses | |  | | | 5.584962501 |
| GRMZM2G138468 | Glycoside hydrolase family 13; | | | defence related | | NP_001105539 | | | 5.584962501 |
| GRMZM2G454550 | Glycoside hydrolase, catalytic core; | | | defence related | |  | | | 4.584962501 |
| GRMZM2G343828 | Glycoside hydrolase, catalytic core; | | | defence related | |  | | | 4.584962501 |
| GRMZM2G108032 | Glycoside hydrolase, family 10; | | | defence related | | NP_001140909 | | | 6.569855608 |
| GRMZM2G055101 | Glycoside hydrolase, family 10; | | | defence related | |  | | | 8.661778098 |
| GRMZM2G041961 | Glycoside hydrolase, family 17; | | | defence related | | NP_001150348 | | | 6.894817763 |
| GRMZM2G123107 | Glycoside hydrolase, family 17; | | | defence related | |  | | | 6.14974712 |
| GRMZM2G125032 | Glycoside hydrolase, family 17; | | | defence related | | NP_001140332 | | | 5.584962501 |
| GRMZM2G144815 | Glycoside hydrolase, family 20; | | | defence related | |  | | | 4.584962501 |
| GRMZM2G071883 | Glycoside hydrolase, family 35; | | | defence related | | NP_001170541 | | | 4.584962501 |
| GRMZM2G132464 | Glycoside hydrolase, family 5, conserved site; | | | defence related | | NP_001169547 | | | 6.14974712 |
| GRMZM2G126468 | Glycoside hydrolase, family 63; | | | defence related | |  | | | 4.584962501 |
| GRMZM2G381782 | Glycoside hydrolase, family 81; | | | defence related | |  | | | 4.584962501 |
| GRMZM2G015143 | Glycoside hydrolase/deacetylase, beta/alpha-barrel; | | | defence related | |  | | | 4.584962501 |
| GRMZM2G453388 | Glucose/ribitol dehydrogenase; | | | defence related | | NP_001167965 | | | 4.584962501 |
| GRMZM2G108338 | Glucose/ribitol dehydrogenase; | | | defence related | | NP_001152614 | | | 4.584962501 |
| GRMZM2G339994 | Glucose/ribitol dehydrogenase; | | | defence related | | NP_001140439 | | | 4.584962501 |
| GRMZM2G389019 | Glucose/ribitol dehydrogenase | | | defence related | |  | | | 4.584962501 |
| GRMZM2G453311 | Glucose/ribitol dehydrogenase; | | | defence related | |  | | | 4.584962501 |
| GRMZM2G076981 | Glucose/ribitol dehydrogenase; | | | defence related | |  | | | 6.569855608 |
| GRMZM2G126496 | Glucose/ribitol dehydrogenase; | | | defence related | |  | | | 5.584962501 |
| GRMZM2G453388 | Glucose/ribitol dehydrogenase; | | | defence related | | NP_001167965 | | | 4.584962501 |
| GRMZM2G108338 | Glucose/ribitol dehydrogenase; | | | defence related | | NP_001152614 | | | 4.584962501 |
| GRMZM2G339994 | Glucose/ribitol dehydrogenase; | | | defence related | | NP_001140439 | | | 4.584962501 |
| GRMZM2G389019 | Glucose/ribitol dehydrogenase | | | defence related | |  | | | 4.584962501 |

**Table S2. The primers used to perform the Real-time PCR in this study.**

| **Primer ID** | **Primer sequences** | **Gene ID** | **Expression trend(Treat/CK)** | **Gene description** | **log2(4-d/2-CK)** |
| --- | --- | --- | --- | --- | --- |
| G-F1 | GCAGCCAGGACTTCTACGAC | GRMZM2G136372 | Up | Thaumatin, pathogenesis-related protein | 9.274 |
| G-R1 | GGCAGAAGGTGACCTGGTAG |  |  |  |  |
| G-F2 | CTCCCCGAACAAGAACAAGA | GRMZM2G123119 | Up | Pathogenesis-related transcriptional factor | 9.896 |
| G-R2 | AATGGAACTCGGATGACACC |  |  |  |  |
| G-F3 | GAAGGTGCTGTGGAGAAAGC | GRMZM2G332390 | Up | Auxin responsive SAUR protein | 10.764 |
| G-R3 | ACCGGCACAAGGACAGTCT |  |  |  |  |
| G-F4 | TGCTGGCATCTTACAGCAAC | GRMZM2G397755 | Up | Helix-loop-helix DNA-binding protein | 11.394 |
| G-R4 | ATCTTGGACAGCACCGACAT |  |  |  |  |
| G-F5 | AGCTCCCAGCATGTTCAGTT | GRMZM2G012242 | Up | Major facilitator superfamily transporter | 10.456 |
| G-R5 | GCTCTCCAGACACTCGAACC |  |  |  |  |
| G-F6 | TTTCCCAGCACTTCTTGACC | GRMZM2G113860 | Up | Zinc finger, C2H2-type protein | 10.033 |
| G-R6 | GGCACCACGACTGCTTATTA |  |  |  |  |
| G-F7 | GGAGCACGTCGGGAAAGGGC | GRMZM2G179896 | Up | terpene synthase 6 | 7.681 |
| G-R7 | GCGCATCTGCACAGCACACT |  |  |  |  |
| G-F8 | GGGAGTTCAGGGACTTGTTCG | GRMZM2G085924 | Down | O-methyltransferase, COMT | -3.75 |
| G-R8 | GGTGGCAATGATCTTGGGAG |  |  |  |  |
| G-F9 | CGCACAAGCTCAGGCTATAC | GRMZM2G059191 | Down | Ferredoxin--NADP reductase | -3.55 |
| G-R9 | AAGCGATACCGTCTTGGAGT |  |  |  |  |
| G-F10 | CGGCTCACCTTTGATTCCT | GRMZM2G151425 | UP | Universal stress protein A | 11.033 |
| G-R10 | CTCACAGAGTTTCTCCCTTGC |  |  |  |  |
| G-F11 | CCAAGGCCGAGGTCTACTAC | GRMZM2G065203 | Down | Phospholipid/glycerol acyltransferase | -3.99 |
| G-R11 | ACTCCGTCAGGTCCTCCA |  |  |  |  |
| G-F12 | TAAGCCAATGGTCGAGACTG | GRMZM2G303419 | Down | SAM dependent carboxyl methyltransferase | -5.98 |
| G-R12 | TGCTCAGGACGCTAGAGATG |  |  |  |  |
